# Atypical cell death and insufficient matrix organization in long-bone growth plates from *Tric-b*-knockout mice

**DOI:** 10.1038/s41419-023-06285-y

**Published:** 2023-12-20

**Authors:** Atsuhiko Ichimura, Yuu Miyazaki, Hiroki Nagatomo, Takaaki Kawabe, Nobuhisa Nakajima, Ga Eun Kim, Masato Tomizawa, Naoki Okamoto, Shinji Komazaki, Sho Kakizawa, Miyuki Nishi, Hiroshi Takeshima

**Affiliations:** 1 Graduate School of Pharmaceutical Sciences, Kyoto University, Kyoto, 606-8501 Japan; 2 Saitama Medical University, Saitama, 350-0495 Japan

**Keywords:** Calcium channels, Apoptosis, Growth disorders, Bone

## Abstract

TRIC-A and TRIC-B proteins form homotrimeric cation-permeable channels in the endoplasmic reticulum (ER) and nuclear membranes and are thought to contribute to counterionic flux coupled with store Ca^2+^ release in various cell types. Serious mutations in the *TRIC-B* (also referred to as *TMEM38B*) locus cause autosomal recessive osteogenesis imperfecta (OI), which is characterized by insufficient bone mineralization. We have reported that *Tric-b*-knockout mice can be used as an OI model; *Tric-b* deficiency deranges ER Ca^2+^ handling and thus reduces extracellular matrix (ECM) synthesis in osteoblasts, leading to poor mineralization. Here we report irregular cell death and insufficient ECM in long-bone growth plates from *Tric-b*-knockout embryos. In the knockout growth plate chondrocytes, excess pro-collagen fibers were occasionally accumulated in severely dilated ER elements. Of the major ER stress pathways, activated PERK/eIF2α (PKR-like ER kinase/ eukaryotic initiation factor 2α) signaling seemed to inordinately alter gene expression to induce apoptosis-related proteins including CHOP (CCAAT/enhancer binding protein homologous protein) and caspase 12 in the knockout chondrocytes. Ca^2+^ imaging detected aberrant Ca^2+^ handling in the knockout chondrocytes; ER Ca^2+^ release was impaired, while cytoplasmic Ca^2+^ level was elevated. Our observations suggest that *Tric-b* deficiency directs growth plate chondrocytes to pro-apoptotic states by compromising cellular Ca^2+^-handling and exacerbating ER stress response, leading to impaired ECM synthesis and accidental cell death.

## Introduction

Ca^2+^ store functions organized in the sarco/endoplasmic reticulum (SR/ER) are essential for cellular homeostasis. Store Ca^2+^ fluxes are predominantly mediated by SR/ER Ca^2+^-ATPase pumps and Ca^2+^ release channels, namely inositol trisphosphate (IP_3_R) and ryanodine receptors (RyR), and supposed to accompany counterion fluxes to balance charge, osmolality and pH between the SR/ER lumen and the cytoplasm [1–3]. Although several ionic fluxes, such as K^+^, Cl^-^ and H^+^ currents, have been detected in SR/ER membranes [4–6], the molecular basis of the counterionic fluxes is largely unknown. It is reasonably proposed that counterion species and their current densities during Ca^2+^ uptake and release are divergent among cell types, because SR/ER-resident channels and transporters may be differentially expressed to generate various counterion fluxes. We previously identified two trimeric intracellular cation channel subtypes, namely TRIC-A and TRIC-B, both of which are distributed to the SR/ER and nuclear membranes and form ionic channels that are predominantly permeable to monovalent cations in planner lipid bilayer membranes [7–9]. The unique three-dimensional structures of TRIC channels have been elucidated, and each subunit possesses its own ion-conducting pore equipped with phospholipids under intracellular conditions [10–12]. In knockout mice lacking TRIC subtypes, cell types developing functional defects commonly exhibit impaired Ca^2+^ release and store Ca^2+^ overloading. For example, *Tric-a*-knockout mice exhibit impaired RyR-mediated Ca^2+^ release in muscle cells [13, 14]. In contrast, IP_3_R-mediated Ca^2+^ release is compromised in alveolar epithelial cells and osteoblasts from *Tric-b*-knockout mice [15, 16]. These observations indicate that TRIC channels generate counter-K^+^ fluxes at least in part to facilitate store Ca^2+^ release in various cells. Additionally, more recent observations suggest that TRIC-A directly interacts with and activates RyR channels in addition to providing a counterion current [17].

Long bones develop through the biological process called endochondral ossification, and the initial stage of this process is cartilage formation [18]. During the cartilage formation in early embryogenesis, chondroblasts undergo differentiation and organize morphologically distinct zones, each of which contains homogeneous chondrocytes specified by morphological characteristics. Round-shaped chondrocytes propagate in the epiphyseal end and produce type II collagen COL2A1. Then, the round chondrocytes structurally change into flat chondrocytes that proliferate to arrange characteristic columnar arrays. The columnar chondrocytes subsequently differentiate into hypertrophic chondrocytes expressing type X collagen, and finally swell and undergo apoptosis. Finally, the region scattered with the generated apoptotic bodies is gradually replaced by trabecular bone through the action of osteoclasts and osteoblasts.

Osteogenesis imperfecta (OI) is a genetic disease characterized by repeated bone fractures due to reduced bone mass [19]. The majority of OI cases result from defective type I collagen; structural mutations and altered posttranslational modifications lead to its insufficient synthesis, unfolding, mistrafficking, poor secretion and disincorporation into the bone matrix. OI-causing mutations are also found in collagen-unrelated genes, such as the osteoblast-specific transcription factor Osterix and the osteoblast-specific transmembrane protein IFITM5. Furthermore, homozygous deletion mutations in the *TRIC-B* (also referred to as *TMEM38B*) locus have been identified in several OI pedigrees [20–23]; the critical mutations are all supposed to produce defective TRIC-B channels in the reported patients. Indeed, *Tric-b*-knockout mice develop an OI-like phenotype, and the *Tric-b* deficiency induces store Ca^2+^ overloading due to compromised Ca^2+^ release in osteoblasts [16]. The experimental evidence indicates that the pro-collagen processing in the ER is likely deranged by the defective store Ca^2+^ handling of osteoblasts, leading to insufficient bone matrix and resulting in poor bone mineralization in OI patients with *TRIC-B* mutations [16, 24]. However, the impact of the *Tric-b* deficiency has not been examined in growth plates contributing to long-bone outgrowth. In this report, we investigated the irregular cell death and impaired extracellular matrix (ECM) synthesis observed in *Tric-b*-knockout growth plate chondrocytes.

## Results

### Dead cells in *Tric-b*-knockout growth plates

**Fig. 1:**
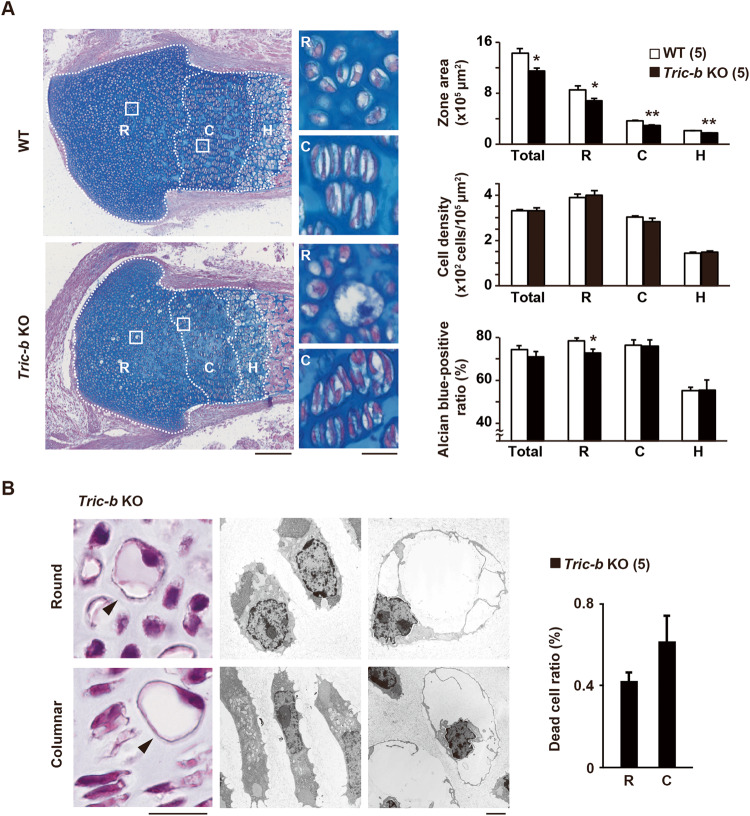
Insufficient ECM organization and aberrant cell death in *Tric-b*-knockout growth plates. **(A)** Zonal area, cell density and ECM occupancy in E18.5 *Tric-b*-knockout growth plates. Alcian blue-stained femoral growth plate sectional images (left images; scale bar, 200 μm). Round (R), columnar (C) and hypertrophic (H) chondrocyte zones are indicated by white dashed lines. Typical round (R) and columnar (C) chondrocytes are also shown in the magnified images (scale bar, 20 µm). The zonal area size, cell density normalized to zonal size and alcian blue-positive portion in total growth plate and individual cell zones are statistically analyzed between the knockout and wild-type growth plates (right bar graphs). (**B**) Photo-microscopic images of dilated dead cells (left panels), electron-microscopic images of apparently normal cells (middle panels) and dilated dead cells (right panels) observed in E18.5 *Tric-b*-knockout round and columnar cell zones. Scale bars, 20 μm. The frequencies of dilated dead round and columnar chondrocytes detected in the knockout growth plates are shown in the bar graph. In the bar graphs, the data are presented as the mean ± SEM., and the numbers of mice examined are shown in parentheses. Statistical differences between the genotypes are indicated by asterisks (*p < 0.05 and **p < 0.01 in *t*-test).

We first explored the impact of *Tric-b* deficiency on growth plate chondrocytes by histologically analyzing developing bones from the *Tric-b*-knockout mice just before birth (E18.5). Consistent with the previous observation that the body size of *Tric-b*-knockout mice is slightly decreased when compared with that of wild-type littermates [15], the femoral length was reduced in the knockout mice (Fig. S1A). In the longitudinal femoral sections prepared from the knockout mice, round, columnar and hypertrophic chondrocyte zones were regularly formed to constitute the developing growth plates, while their respective areas were decreased when compared with wild-type controls (Fig. 1A). The knockout and wild-type growth plate chondrocytes were roughly similar in morphology, and cell densities normalized to each zonal area were also similar between the genotypes. However, alcian blue staining for acidic proteoglycans detected that the ECM portion of the round chondrocyte zone was significantly decreased in *Tric-b*-knockout growth plates.

Unexpectedly, *Tric-b*-knockout growth plates contained severely dilated cells, which were largely negative in hematoxylin-eosin staining and randomly located in the round and columnar cell zones (Fig. 1B). In electron-microscopic observation, the dilated cells contained highly condensed nuclei and almost empty cytoplasm indicative of apoptosis. Although the dilated dead cells were very low in frequency (~0.6% of total cells in combined round and columnar cell area), such irregular cells were never detected in wild-type femoral bones. The dead cells were also detected in the growth plates of humeral and rib bones isolated from the knockout mice (Fig. S1B). TdT-mediated dUTP-biotin nick end labelling (TUNEL) has been used for detecting DNA fragmentation in apoptotic cells. Although TUNEL-positive cells could be hardly detected due to their low numbers, the positive cells were significantly increased in *Tric-b*-knockout round and columnar cell zones (Fig. S2A). Therefore, *Tric-b* deficiency seemed to occasionally cause apoptotic cell death in proliferating growth plate chondrocytes during long bone development. On the other hand, proliferating cell nuclear antigen (PCNA) is an auxiliary component of DNA polymerase during DNA duplication and repair, and has been used as a marker for proliferating cells. PCNA-positive cells were observed with similar frequencies between *Tric-b*-knockout and wild-type growth plates, suggesting that *Tric-b* deficiency does not obviously affect cell cycle in proliferating chondrocytes (Fig. S2B).

### Pro-collagen accumulation in *Tric-b*-knockout chondrocytes

**Fig. 2:**
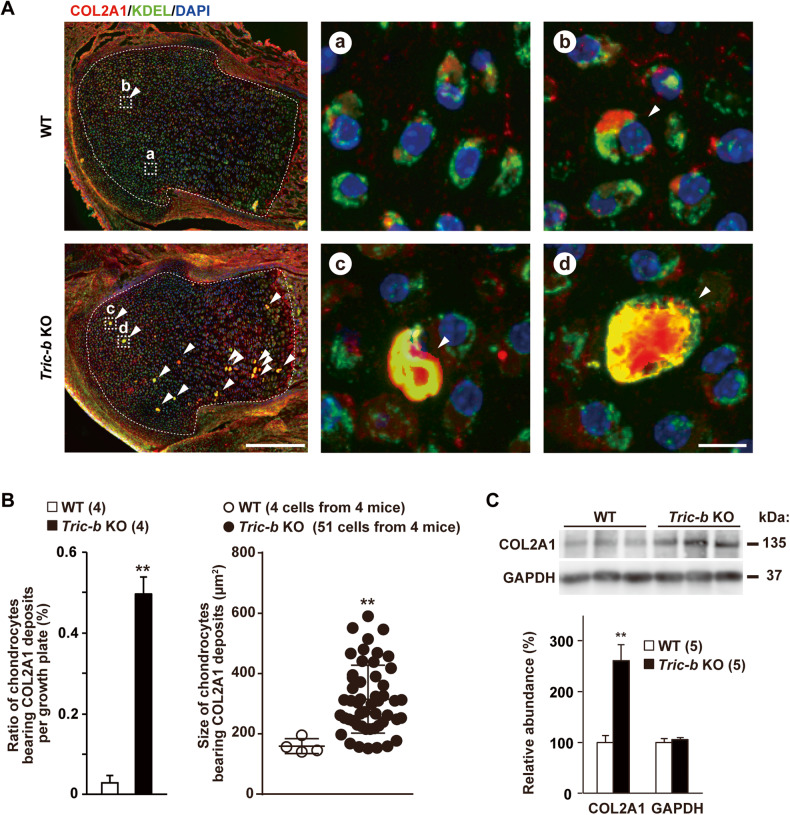
Pro-collagen overaccumulation in *Tric-b*-knockout round chondrocytes. (**A**) Confocal microscopic images of round chondrocytes, nuclear-stained with DAPI and fluorescence-stained using antibodies against COL2A1 and the ER marker KDEL. As seen in the left low-magnification images (scale bar, 200 μm), COL2A1-positive intracellular deposits (>140 μm^2^, arrowheads) were frequently detected in the round cell zones of *Tric-b*-knockout growth plates. The growth plate chondrocyte zones are indicated by the white dashed lines, and high-magnification images of the cells presenting are indicated by the white dashed boxes. In the high-magnification images (scale bar, 10 μm), wild-type growth plate panels show regular round chondrocytes (a) and one COL2A1 deposit-bearing cell (b), while *Tric-b*-knockout growth plate panels show dilated cells developing COL2A1 deposits (c, d). (**B**) Appearance ratios and cell sizes of COL2A1-positive chondrocytes in growth plates. (**C**) Western blot analysis of intracellular COL2A1 in growth plate chondrocytes. Representative COL2A1-immunoreactivities were shown (upper panel), and the digitalized immunoreactivities are statistically compared between wild-type and *Tric-b*-knockout round chondrocytes (lower bar graph). Glyceraldehyde-3-phosphate dehydrogenase (GAPDH) was used as a loading control. In the bar and scatter graphs, the data are presented as the mean ± SEM, and the numbers of mice examined are shown in parentheses. Statistical differences from the genotypes are indicated by asterisks (**p < 0.01 in *t*-test).

Proliferating growth plate chondrocytes mainly produce COL2A1 as a major cartilage matrix component. We next focused on collagen synthesis in *Tric-b*-knockout chondrocytes. In wild-type round chondrocytes from developing femoral bones, COL2A1 deposits occasionally appeared as intracellular puncta (>5 μm^2^) without colocalization with the ER maker KDEL sequence (Fig. 2A upper panels). However, in *Tric-b*-knockout chondrocytes, COL2A1 deposits were more frequently detected and colocalized with the ER marker (Fig. 2B). Furthermore, the deposits became larger and denser in *Tric-b*-knockout growth plates, and such severely expanded deposits sporadically covered the bulk of cytoplasm in the presumed dying cells (Fig. 2A lower panels).

To assess the relative amount of intracellular COL2A1 within growth plate chondrocytes, we solubilized intracellular proteins in a deoxychorate-containing solution and removed ECM by centrifugation (Fig. 2C). *Tric-b*-knockout lysates reproducibly exhibited dense immunostaining signals against COL2A1, although signals against the control glycolytic enzyme were similar between knockout and wild-type lysates. The results, together with the histochemical observations, indicated that the ER elements were overloaded with COL2A1 fibers in *Tric-b*-knockout chondrocytes. It is reasonably proposed that *Tric-b* deficiency deranges the ER processing or ER-Golgi trafficking of the pro-collagen fibers.

### ER stress in *Tric-b*-knockout chondrocytes

**Fig. 3:**
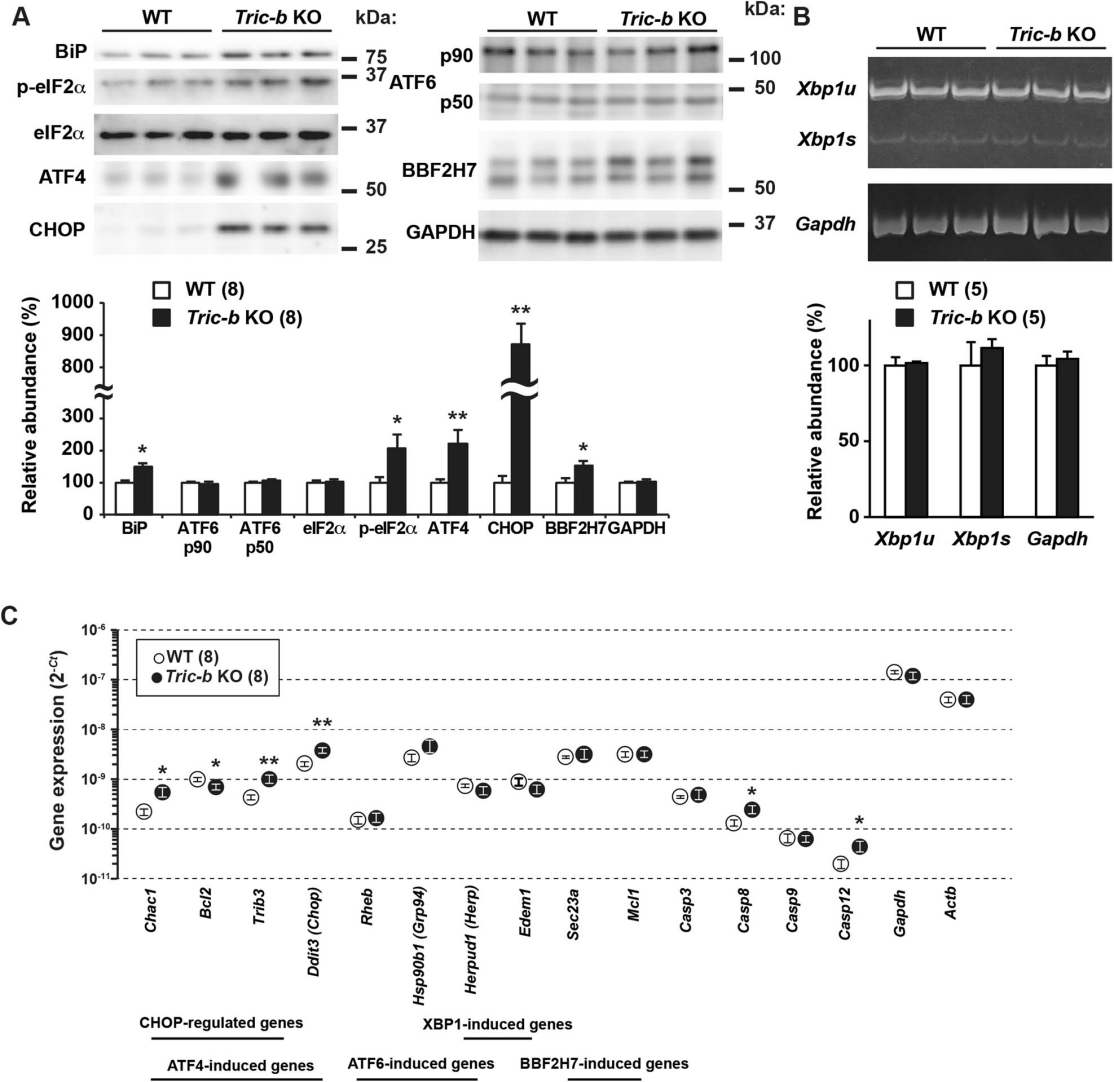
Altered ER stress responses in *Tric-b*-knockout chondrocytes. (**A**) Western blot analysis of UPR-related proteins in cell lysates prepared from round chondrocyte-enriched growth plates. Target protein contents were monitored using antibodies against BiP, phospho- and total-eIF2α, ATF4, CHOP, p90 and p50 forms of ATF6 and BBF2H7. Representative immunoblot images are shown in the upper panels, and relative immunoreactivities normalized to wild-type values are presented in the bar graph. GAPDH was used as a loading control. (**B**) RT-PCR analysis monitoring the unspliced (*Xbp1u*) and spliced (*Xbp1s*) *Xbp1* mRNAs in round chondrocyte-enriched growth plates. Electrophoresis gel images of amplified cDNAs are shown in the upper panel, and relative cDNA intensities normalized to wild-type values are presented in the bar graph. *Gapdh* mRNA was also amplified as an internal control. (**C**) Quantitative RT-PCR analysis monitoring mRNAs transcribed from UPR-related genes in round chondrocyte-enriched growth plates. The cycle threshold (*Ct*) indicates the cycle number at which the amount of amplified cDNA reaches a fixed threshold in each RT-PCR reaction. In the graphs, the data are presented as the mean ± SEM, and the numbers of mice examined are shown in parentheses. Statistical differences from the genotypes are indicated by asterisks (*p < 0.05 and **p < 0.01 in *t*-test).

When immature protein levels reach maximal acceptable levels in the ER lumen, the major transmembrane sensors IRE1 (transmembrane protein kinase inositol-requiring enzyme 1), ATF6 (activating transcription factor 6) and PERK (PKR-like ER kinase) activate the unfolded protein response (UPR) as a homeostatic mechanism in response to ER stresses [25]. For example, the ER chaperone BiP/GRP78 is susceptibly induced by activation of either the sensor proteins in various cell types. Under physiological conditions, UPR is moderately activated in cell types that abundantly produce secretory proteins, such as growth plate chondrocytes, pancreatic β cells and plasma cells that extensively produce collagen, insulin and antibodies, respectively. To investigate atypical UPR levels in *Tric-b*-knockout chondrocytes, we prepared cell lysates and total RNA from the round chondrocyte-enriched femoral epiphyses. In Western blot analysis, BiP contents were higher in the knockout lysates than those in wild-type lysates, indicating that UPR was highly activated in *Tric-b*-knockout chondrocytes (Fig. 3A).

Activated PERK phosphorylates the eukaryotic translation initiation factor eIF2α to reduce translation of newly synthesized proteins and to selectively induce the transcription factor ATF4 for the induction of stress-related genes such as *Chac1* and *Trb3* [25, 26]. Western blot analysis showed that both phospho-eIF2α and ATF4 contents were remarkably elevated in the knockout lysates (Fig. 3A). RT-PCR analysis indicated that *Chac1* and *Trib3* mRNAs were inducibly transcribed in the knockout chondrocytes (Fig. 3C). Therefore, PERK/eIF2α signaling seemed to be highly activated in the knockout chondrocytes. This conclusion was further supported by the immunohistological staining of ATF4 and COL2A1 (Fig. S3). ATF4 immunofluorescence was positive in ~30 cells per wild-type growth plate, while ~106 ATF4-positive cells were detected in the *Tric-b*-knockout growth plate. When focusing on the cell populations bearing large COL2A1 deposits (>10 μm^2^), ~5 cells were assigned as ATF4-COL2A1 deposit-double positive cells in the wild-type growth plate, while such double-positive cells were increased to ~50 cells in the knockout growth plate.

IRE1 activation stimulates *Xbp1* mRNA splicing and thus promotes the transcription of XBP1-induced genes including *Edem1* and *Erdj4* [27]. In RT-PCR analysis, *Tric-b*-knockout growth plates exhibited no aberrant features in *Xbp1* mRNA splicing and XBP1-induced gene expression (Fig. 3B, C). On the other hand, ATF6 p90 is cleaved under ER stress conditions, and the resulting cytoplasmic fragment ATF6 p50 translocates into the nucleus as an active transcription factor to induce the expression of the ATF6-induced genes, including *Grp94* and *Herp* [28]. We observed similar ATF6 cleavage and ATF6-induced gene expression between *Tric-b*-knockout and wild-type specimens (Fig. 3B, C). Therefore, of the major UPR pathways, IRE1 and ATF6 signalings were functioning at similar intensities between the knockout and wild-type chondrocytes, while PERK/eIF2α signaling seemed to be facilitated in the knockout cells. This conclusion was further supported by the microarray data derived from round chondrocyte-enriched specimens (Fig. S4A); the heatmap data suggested that CHOP-regulated and ATF4-induced genes were preferentially activated presumably downstream of eIF2α phosphorylation in the knockout chondrocytes.

BBF2H7 (box B-binding factor 2 human homolog on chromosome 7) is an ER stress sensor essential for chondrogenesis, and its cleaved activation stimulates *Sec23a* and *Mcl1* gene expression [29]. SEC23A protein contributes to COPII vesicle formation for ER-Golgi trafficking, while MCL1 (myeloid cell leukemia sequence 1) belongs to the anti-apoptotic BCL-2 (B-cell leukemia/lymphoma 2) family. Impaired *Sec23a* and *Mcl1* expression promotes ER dilation and apoptosis in *Bbf2h7*-knockout chondrocytes [29], similar to the morphological abnormalities observed in *Tric-b*-knockout chondrocytes. Western blotting results showed that BBF2H7 was cleaved more than normal in the knockout chondrocytes (Fig. 3A), and RT-PCR analysis indicated that both *Sec23a* and *Mcl1* genes were similarly activated between the knockout and wild-type chondrocytes (Fig. 3C). Therefore, the BBF2H7 pathway seemed to function normally in *Tric-b*-knockout chondrocytes.

### Apoptosis-related alterations in *Tric-b*-knockout chondrocytes

**Fig. 4:**
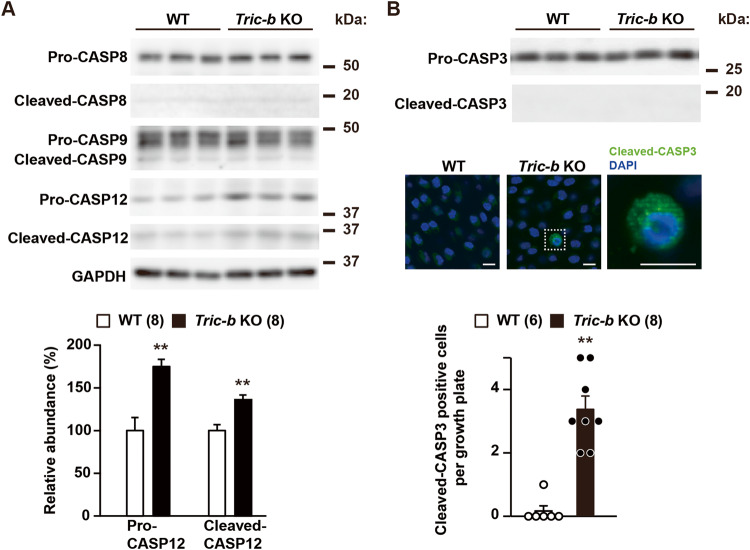
Caspase subtypes in *Tric-b*-knockout chondrocytes. (**A**) Western blot analysis of CASP8, CASP9 and CASP12 in cell lysates prepared from round chondrocyte-enriched growth plates. Both pro- and cleaved forms of the caspase subtypes were detected by specific antibodies, and representative immunoreactivities are presented (upper panels). The immunoreactivities of pro- and cleaved-CASP12 were digitalized and are statistically compared between wild-type and *Tric-b*-knockout specimens (bar graph). GAPDH was also analyzed as an internal control. (**B**) Immunochemical analysis of CASP3. Western blot analysis in round chondrocyte-enriched lysates indicated similar pro-CASP3 levels in wild-type and *Tric-b*-knockout specimens, but failed to detect cleaved-CASP3 in both the specimens (upper panels). However, immunohistochemical analysis occasionally detected cleaved-CASP3-positive cells in *Tric-b*-knockout round chondrocyte zones (middle panels: scale bar, 10 μm). The appearance frequencies of cleaved-CASP3-positive cells were statistically compared between wild-type and *Tric-b*-knockout growth plates (bar graph). In the bargraphs, the data are presented as the mean ± SEM., and the numbers of mice examined are shown in parentheses. Statistical differences from the genotypes are indicated by asterisks (**p < 0.01 in *t*-test).

It has been reported that caspase 8 (CASP8), CASP9 and CASP12 become differentially active to function as initiator caspases under severe ER stress conditions [30]. Western blotting suggested no aberrant activation of CASP8 and CASP9 in *Tric-b*-knockout chondrocytes (Fig. 4A). In contrast, both intact and cleaved forms of CASP12 were more abundant in the knockout growth plates than in wild-type controls. Therefore, of the ER stress-related caspase subtypes, CASP12 was most likely preferentially activated in *Tric-b*-knockout chondrocytes.

CASP3 catalyzes the cleavage of many key cellular proteins and serves as a major effector caspase that executes apoptosis [30]. Western blotting failed to detect cleaved-CASP3 in both *Tric-b*-knockout and control growth plates (Fig. 4B). However, immunohistochemical analysis occasionally detected cleaved CASP3-positive dilated cells in *Tric-b*-knockout growth plates, while such immunofluorescence-positive cells were never observed in wild-type growth plates (Fig. 4B). Therefore, the cleaved CASP3-positive cells were probably assigned as dilated chondrocytes undergoing apoptosis in *Tric-b*-knockout growth plates. Overall, CASP12 and CASP3 likely contributed to *Tric-b*-knockout apoptosis as initiator and effector caspases, respectively.

### Altered store Ca^2+^ handling in *Tric-b*-knockout chondrocytes

**Fig. 5:**
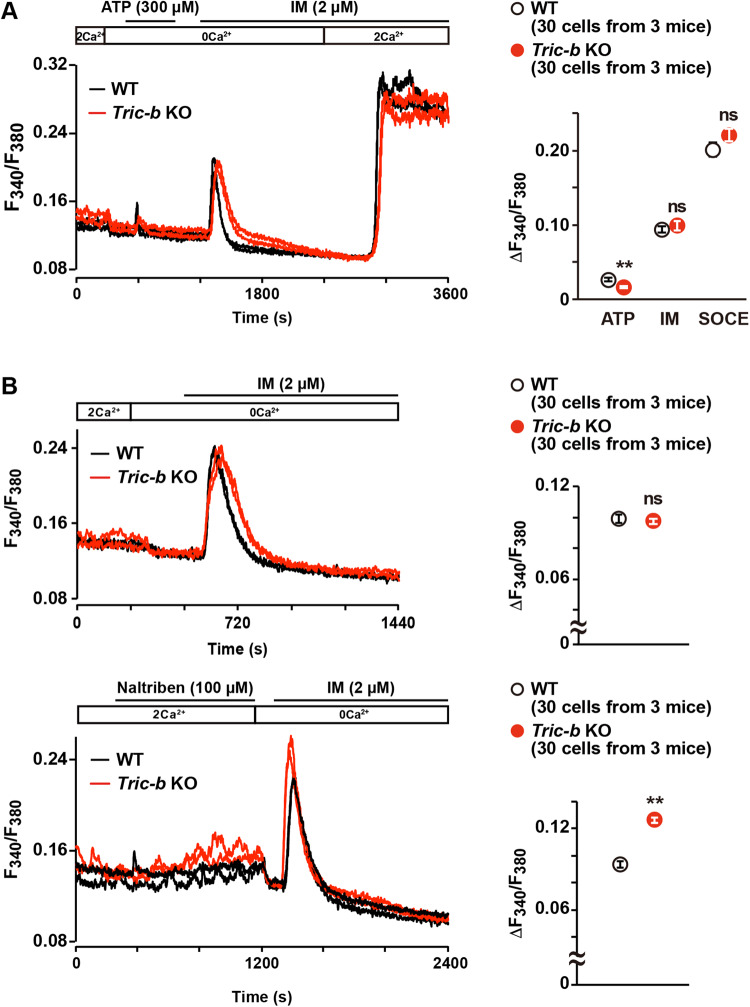
Impaired store Ca^2+^ handling in *Tric-b*-knockout chondrocytes. Round chondrocytes in E17.5 femoral bone slices were examined by Fura-2 imaging. (**A**) Using the perfusion protocol indicated, representative recording traces obtained from two cells in wild-type (WT) and *Tric-b* knockout mice are shown (left panel). ATP-evoked Ca^2+^ transients (ATP), ionomycin-induced Ca^2+^ leak responses (IM) and store-operated Ca^2+^ entry responses (SOCE) are statistically analyzed (dot graph). (**B**) Ionomycin-induced Ca^2+^ responses with or without naltriben pretreatment. Representative recording traces from two cells in each genotype are shown (left panels), and the observed Ca^2+^ responses were statistically analyzed (dot graphs). The data are presented as the mean ± SEM, and the numbers of cells and mice examined are shown in parentheses. Statistical differences between the genotypes are marked with asterisks (**p < 0.01 in *t*-test). ns not significant.

In our previous studies, impaired store Ca^2+^ release and store Ca^2+^ overloading were commonly observed in functionally defective cells prepared from *Tric-a*- and *Tric-b*-knockout mice [9, 13–15]. To examine store Ca^2+^ handling of *Tric-b*-knockout chondrocytes, we prepared slice specimens from embryonic femoral bones and collected Fura-2 imaging data from round chondrocytes. Using a perfusion protocol with normal, Ca^2+^-free, ATP-supplemented and Ca^2+^ ionophore ionomycin-containing bathing solutions (Fig. 5A), we examined IP_3_-induced Ca^2+^ release in response to purinergic P2Y receptor activation, ionomycin-induced Ca^2+^ leak and store-operated Ca^2+^ entry (SOCE). In *Tric-b*-knockout chondrocytes, Ca^2+^ transients evoked by P2Y receptor activation became weak, but ionomycin-induced Ca^2+^ leak and SOCE remained unaltered (Fig. 5A). Therefore, IP_3_-induced Ca^2+^ release was significantly impaired in the knockout chondrocytes. However, it was rather surprising that intracellular stores were not Ca^2+^-overloaded despite the impaired IP_3_R-mediated Ca^2+^ release in the knockout chondrocytes (Fig. 5B).

Growth plate chondrocytes generate spontaneous Ca^2+^ influx by intermissive gating of cell-surface TRPM7 channels, which can be pharmacologically activated with the channel agonist naltriben [31]. Thus, it is presumed that naltriben preconditioning facilitates autonomic Ca^2+^ entry and ensures full Ca^2+^ loading in intracellular stores. Immediately after naltriben pretreatment, ionomycin-induced Ca^2+^ release was obviously higher in *Tric-b*-knockout chondrocytes than in wild-type controls (Fig. 5B). Therefore, *Tric-b* deficiency might decelerate Ca^2+^ leakage from full stores, thus generating temporal Ca^2+^-overloaded stores. The temporal Ca^2+^ overloading and weakened ATP-induced Ca^2+^ release were consistent with the prevailing notion that TRIC channels facilitate Ca^2+^ release by providing counter-cationic currents [9]. It might be a reasonable speculation that IP_3_R gating is enhanced, thus facilitating Ca^2+^ leakage and preventing store Ca^2+^ overloading in the knockout chondrocytes, because steady-state phospholipase C (PLC) activity was likely elevated under PERK-activated conditions (see below section).

### Facilitated Ca^2+^ entry in *Tric-b*-knockout chondrocytes

**Fig. 6:**
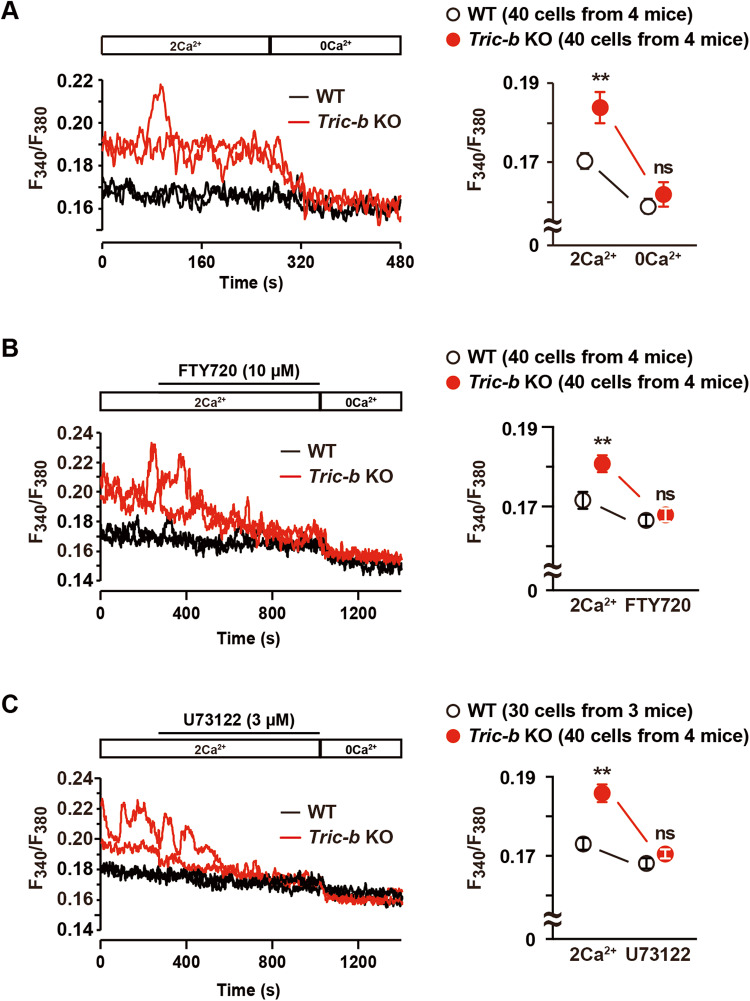
Elevated resting [Ca^2+^]_i_ in *Tric-b*-knockout chondrocytes. In the Fura-2 imaging using the perfusion protocol indicated, representative recording traces obtained from two cells in wild-type (WT) and *Tric-b* knockout mice are shown (left panels). The effects of Ca^2+^-free bathing solution (**A**), the TRPM7 inhibitor FTY720 (**B**) and the PLC inhibitor U73122 (**C**) on resting [Ca^2+^]_i_ are summarized (dot graphs). The data are present as the mean ± SEM, and the numbers of cells and mice examined are shown in parentheses. Statistical differences between the genotypes are marked with asterisks (**p < 0.01 in *t*-test). ns not significant.

The elevation of resting intracellular Ca^2+^ concentration ([Ca^2+^]_i_) is generally associated with enhanced Ca^2+^ influx. In growth plate chondrocytes, TRPM7 channels are intermittently activated by intrinsic phosphoinositol turnover and predominantly responsible for resting Ca^2+^ influx [31]. The resting [Ca^2+^]_i_ of *Tric-b*-knockout chondrocytes was significantly elevated in a normal bathing solution (Figs. 5A and 6A). However, under Ca^2+^-free, TRPM7 inhibitor FTY720-treated and PLC inhibitor U73122-supplemented conditions, *Tric-b*-knockout and wild-type chondrocytes exhibited similar resting [Ca^2+^]_i_ levels (Fig. 6A-C). Therefore, steady-state PLC activity was probably enhanced, and thus, TRPM7-mediated Ca^2+^ entry was facilitated in *Tric-b*-knockout chondrocytes.

**Fig. 7:**
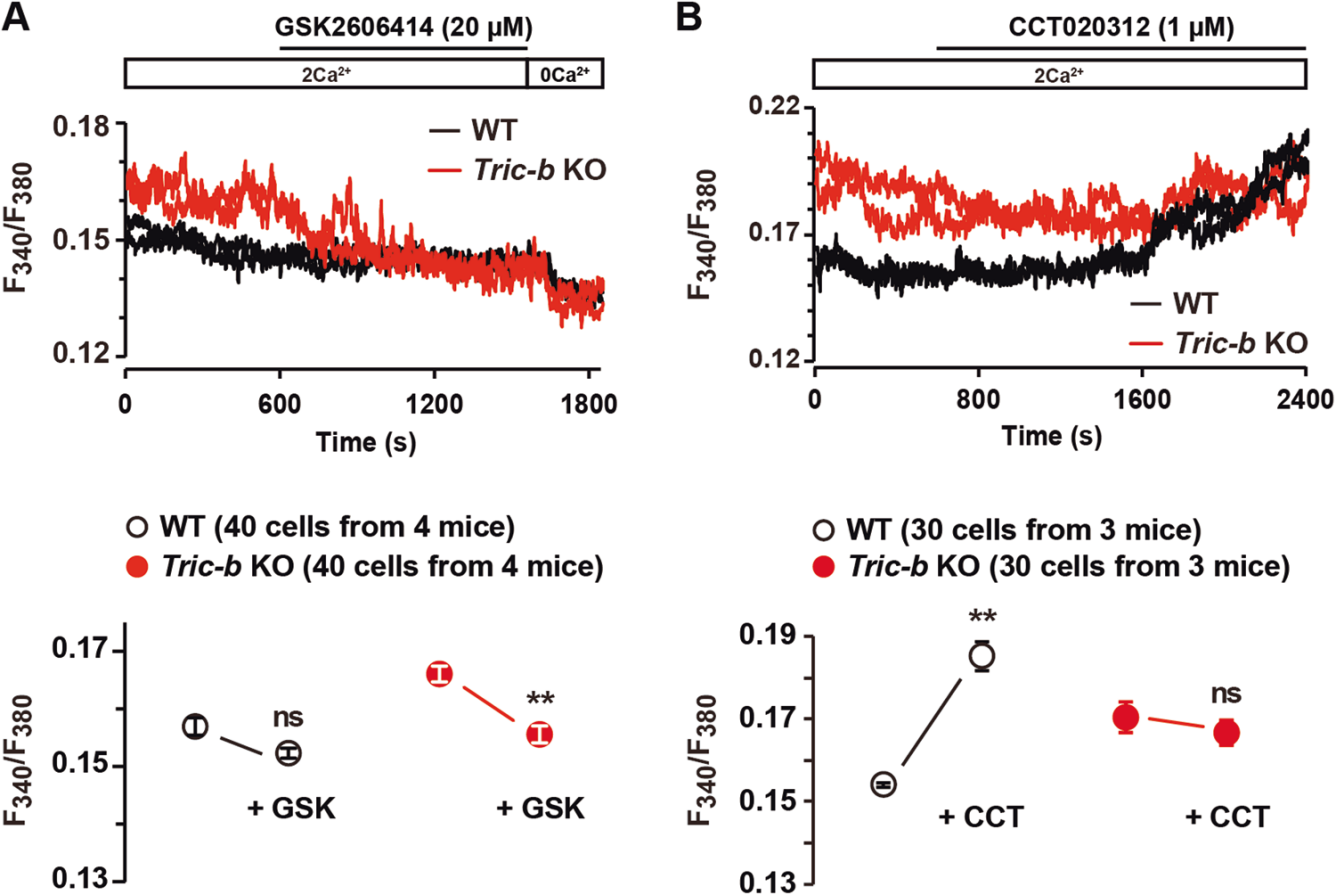
Effects of PERK modulators on resting [Ca^2+^]_i_ in *Tric-b*-knockout chondrocytes. In the Fura-2 imaging using the perfusion protocol indicated, representative recording traces obtained from two cells in wild-type (WT) and *Tric-b* knockout mice are shown (upper panels). The effects of the PERK inhibitor GSK2606414 (**A**) and the PERK activator CCT020312 (**B**) on resting [Ca^2+^]_i_ are summarized (dot graphs). The data are presented as the mean ± SEM, and the numbers of cells and mice examined are shown in parentheses. Statistical differences between resting Fura-2 ratios before and after drug treatments are marked with asterisks (**p < 0.01 in *t*-test). ns not significant.

To evaluate the link between activated PERK/eIF2α signaling and elevated resting [Ca^2+^]_i_ in *Tric-b*-knockout chondrocytes, we utilized the PERK inhibitor GSK2606414 and the PERK activator CCT020312. GSK2606414 treatments (20 μM) did not significantly affect resting [Ca^2+^]_i_ in wild-type chondrocytes but clearly decreased [Ca^2+^]_i_ in the knockout chondrocytes (Fig. 7A). Therefore, under GSK2606414-treated conditions, the knockout and wild-type cells exhibited similar resting [Ca^2+^]_i_ In contrast, CCT020312 treatments (1 μM) obviously elevated [Ca^2+^]_i_ in wild-type chondrocytes but not in the knockout chondrocytes (Fig. 7B). The observations likely suggested that activated PERK/eIF2α signaling mainly contributed to TRPM7 channel facilitation by stimulating steady-state phosphoinositol turnover in the knockout chondrocytes.

## Discussion

OI patients with *TRIC-B* mutations commonly display short stature [20–23], and *Tric-b*-knockout mice are small in body size [15]. Long-bone outgrowth primarily depends on vital proliferation and devoted ECM synthesis in growth plate chondrocytes [18]. Based on the observations in this study, we propose the sequential mechanisms underlying bone outgrowth disturbance and apoptosis in *Tric-b*-knockout growth plates (see the conceptional steps (1) ~ (10) in Fig. S5A). Ca^2+^-dependent chaperones and processing enzymes contribute to the maturation of secretory proteins in the ER and Golgi [32], and vesicular trafficking between the ER and Golgi requires Ca^2+^-dependent processes [33]. Defective store Ca^2+^ handling generally aggravates ER stress by disturbing protein processing and vesicular trafficking; for example, the store Ca^2+^ pump inhibitor thapsigargin is a potent UPR inducer. (step 1) *Tric-b* deficiency compromises store Ca^2+^ handling (Fig. 5) and likely deranges the processing and trafficking machinery, leading to (step 2) the overaccumulation of unfolded proteins including pro-collagen within ER elements in growth plate chondrocytes (Fig. 2). (step 3) Pro-collagen overaccumulation likely aggravates ER stress in *Tric-b*-knockout chondrocytes, and thus (step 4) leads to excess PERK-mediated eIF2α phosphorylation (Fig. 3). The intracellular pro-collagen deposits suggest defective ECM maturation in the ER, while eIF2α hyperphosphorylation likely broadly attenuates cellular translation. Therefore, (step 5) ECM synthesis/secretion is probably impaired in the knockout chondrocytes, and (step 6) ECM contents are reduced in *Tric-b*-knockout growth plates. In particularly, the ECM portion of the round chondrocyte zone is clearly reduced (Fig. 1A), and this regression is likely to mainly contribute to the impaired outgrowth of developing bones in the knockout mice. On the other hand, (step 7) activated PERK/eIF2α signaling seems to induce ATF4 and CHOP expression then lead to excess UPR gene expression (Fig. 3) and (step 8) also may essentially contribute to PLC stimulation for facilitating Ca^2+^ entry and elevating resting [Ca^2+^]_i_ in the knockout chondrocytes (Fig. 7). Accordingly, the knockout chondrocytes may tend to be sensitive to apoptosis, because the calpain-CASP12-CASP3 cascade has been reported in various types of apoptosis [34–36]. In the knockout chondrocytes, (step 9) further incidental [Ca^2+^]_i_ elevation may be predisposed to activate Ca^2+^-dependent calpain for CASP12-cleaved stimulation, and (step 10) resulting CASP12 activation likely triggers the CASP3-mediated apoptotic cascade. However, the accidental apoptosis rarely takes place in knockout growth plates (Figs. 1 and S2) and seems unlikely to obviously contribute to the impaired bone outgrowth.

In conventional processing, the three major ER stress sensors PERK, ATF6 and IRE1 become active by the dissociation of the ER chaperone BiP/GRP78 on the luminal side because excess unfolded proteins attract the chaperone [25]. Our biochemical, gene expression and Ca^2+^ imaging data suggested that ATF6 and IRE1 signalings were similarly activated between *Tric-b*-knockout and wild-type cells, but that PERK/eIF2α signaling was excessively activated in the knockout chondrocytes. To verify the proposed PERK activation, we examined its autophosphorylation by immunoblot analysis, and unexpectedly detected comparable phospho-PERK contents between *Tric-b*-knockout and wild-type growth plates (Fig. S4B). In addition to PERK, three other kinases, HRI, PKR and GCN2, become active under stress conditions such as viral infection and heme depletion, and are known to catalyze eIF2α phosphorylation that initiates PERK/eIF2α signaling [37]. Again, the autophosphorylated forms of the additional kinases seemed similar in content between the genotypes (Fig. S4C). Therefore, eIF2α hyperphosphorylation may not be due to kinase activation, and it can be thus hypothesized that the dephosphorylation of phospho-eIF2α is dampened in the knockout chondrocytes. Previous studies have indicated that the eIF2α dephosphorylation is mainly catalyzed by protein phosphatase 1 (PP1) complexes in several cell types [38] and also that forceful PERK/eIF2α signaling stimulates PP1-mediated eIF2α dephosphorylation as a feedback regulation [39]. A PP1 holoenzyme consists a catalytic subunit, PP1α, PP1β or PP1γ, and one or two regulatory PP1-interacting proteins; >200 distinct PP1 complexes are thought to differentially formed depending on cell types because there are many PP1-interacting proteins so far identified [38]. Therefore, it can be reasonably presumed that PP1-mediated catalytic activity or feedback machinery may be compromised in *Tric-b*-knockout chondrocytes (Fig. S5B). Gene expression profiles for the PP1 catalytic subunits were not altered in the knockout growth plates (Fig. S4A), but the composition of major PP1 complexes is totally unknown in growth plate chondrocytes. To clarify the detailed pathophysiological mechanism underlying the excess activation of PERK/eIF2α signaling in *Tric-b*-knockout chondrocytes, it seems important to elicit the structure and function of PP1 complexes in growth plate chondrocytes.

Of the three major ER stress pathways, the PERK/eIF2α signaling has been repeatedly reported to be linked with aberrant cellular Ca^2+^ handling [40–44]. For example, the PERK/eIF2α signaling is sensitively activated in response to ER Ca^2+^ depletion [40, 41], and elevated [Ca^2+^]_i_ activates PERK/eIF2α signaling to promote apoptosis in virus-infected cells [44]. Such observations may imply that deranged Ca^2+^-handling preferentially induces PERK/eIF2α signaling (see step 3 in Fig. S5A). Furthermore, in our Ca^2+^ imaging experiments, the PERK inhibitor seemed to immediately attenuate activated Ca^2+^ influx in the knockout chondrocytes, suggesting that downstream of activated PERK/eIF2α signaling, PLC is activated for enhancing TRPM7-mediated Ca^2+^ influx in growth plate chondrocytes [31]. Therefore, we can propose a bidirectional link between PERK/eIF2α signaling and cellular Ca^2+^-handling in growth plate chondrocytes; PERK/eIF2α signaling is activated by deranged store Ca^2+^ handling due to *Tric-b* deficiency, and activated PERK/eIF2α signaling stimulates Ca^2+^ influx in a PLC-dependent manner. However, in the PERK/eIF2α signaling cascade proposed thus far, Ca^2+^-dependent processes and Ca^2+^-handling proteins serving as PERK substrates have not been reported. From a biological point of view, the molecular mechanism underlying the proposed bidirectional link seems to be the important issue that needs to be solved in future studies.

## Materials and methods

### Histological analyses

*Tric-b* knockout mice were generated and genotyped as described previously [9].

For histological analysis, the E18.5 femur, humerus and rib bones were fixed in 4% paraformaldehyde, embedded in Super Cryoembedding Medium (Section-lab, Japan), and frozen in liquid nitrogen. Serial cryosections (~10 μm in thickness) were prepared from the fixed specimens and treated with a commercial hematoxylin and eosin solution (Wako Pure Chemical, Japan) or alcian blue solution (Merck, USA) for microscopic observation (BZ-X710, Keyence Co., Japan).

For immunohistochemical analysis, the femoral cryosections were treated with 1% bovine serum albumin to block nonspecific binding. The cryosections were incubated with primary antibodies against COL2A1, ATF4 and KDEL, and then were incubated with an AlexaFluor 488-conjugated antibody against goat anti-mouse IgG and an AlexaFluor 555-conjugated antibody against rabbit IgG (Table S1). After DAPI nuclear staining, fluorescence-labeled sections were examined under a microscope (BZ-X710, Keyence Co). For immunohistochemical analysis of PCNA, the cryosections (4 µm in thickness) were immersed with a citrate buffer and heated at 90 ˚C for 20 min. After treatments with a 3% H_2_O_2_ solution for 5 min and a blocking solution (Blocking One Histo, Nacalai Tesque, Japan) for 30 min, the cryosections were incubated with primary antibodies against PCNA, and then incubated with horseradish peroxidase-conjugated secondary antibody. PCNA reactivity was visualized using diaminobenzidine hydrochloride substrate kit (Abcam, UK) and analyzed by microscopic observation (BZ-X710, Keyence Co.). For TUNEL staining, the cryosections (6 µm in thickness) were stained using TUNEL assay kit (Abcam, ab206386) according to the manufacturer’s instructions and observed by a microscope (BZ-X710, Keyence Co.). Captured images from hematoxylin/eosin-stained and fluorescence-labeled sections were quantitatively analyzed using BZ-X analyzer (Keyence) and ImageJ (U.S. National Institutes of Health) software.

### Ultrastructural analysis

For electron-microscopic analysis, the femoral bones were fixed in prefixative solution (3% paraformaldehyde, 2.5% glutaraldehyde, 0.1 M sodium cacodylate, pH 7.5) and placed in postfixative solution (0.1% OsO_4_, 0.1 M potassium ferricyanide, 0.1 M sodium cacodylate, pH 7.4) at room temperature. The specimens were dehydrated using ethanol and acetone, and embedded in Epon to prepare thin sections (100 ~ 150 nm in thickness) for analysis under a transmission electron microscope (JEM-200CX, JEOL, Japan).

### Gene expression analysis

Total RNA was prepared from mouse tissues using a commercial kit (Isogen, Nippon Gene). RNA preparations from femoral cartilage plate sections enriched with round chondrocytes were reverse-transcribed and analyzed using the GeneChip Mouse Genome 430 2.0 (Affymetrix) according to the manufacturer’s instructions by an outsourcing company (Takara Bio Co., Japan). The array probe intensities were analyzed with the robust multiarray analysis expression algorithm, which represents the log transformation of intensities (background corrected and normalized) from the gene chips [45], and were visualized in the heatmaps.

To further analyze gene expression, mRNA contents were determined by quantitative RT-PCR as described previously [46]. Total RNA was reverse-transcribed using the ReverTra ACE qPCR-RT Kit (Toyobo), and the resulting cDNA was examined by real-time PCR (LightCycler 480 II, Roche). The cycle threshold (*Ct*) was determined from the amplification curve as an index for relative mRNA level in each reaction. The RT-PCR primer sets used in this study are listed in Table S2.

### Immunoblot analysis

Round chondrocyte-enriched growth plate parts were isolated from the E18.5 femoral bones and homogenized in lysis buffer containing 4% sodium deoxycholate, 20 mM Tris-HCl (pH 8.8), 100 mM NaF, 10 mM Na_3_PO_4_, 1 mM Na_3_VO_4_, and 20 mM β-glycerophosphate. After sonication (Astrason Ultrasonic Processor XL, Misonix), the homogenates were centrifuged (17,000 x *g*, 30 min) to remove tissue debris. After measurement of total protein concentration (BCA Protein Assay Kit, Pierce), the resulting growth plate lysates were subjected to 8–16.5% SDS–polyacrylamide gel electrophoresis, and separated proteins were then transferred to PVDF membranes (polyvinylidene difluoride, Merck Millipore). After treatments with a blocking reagent (Blocking One solution, Nacalai Tesque, Japan), the membranes were incubated with primary antibodies and then incubated with secondary antibodies; antibodies were diluted in Can Get Signal (Toyobo, Japan) before the immunoreaction. Immunoreactivity was visualized using a chemiluminescence reagent (GE Healthcare Life Sciences) and an image analyzer (Amersham Imager 600, GE Healthcare Life Sciences) and were quantitatively analyzed using ImageJ software. The antibodies used in this study are listed in Table S1.

### Fura-2 Ca^2+^ imaging

Bone slices were prepared and Ca^2+^ imaging was performed as described previously [31]. The bathing solution used was HEPES-buffered saline (150 mM NaCl, 4 mM KCl, 1 mM MgCl_2_, 2 mM CaCl_2_, 5.6 mM glucose and 5 mM HEPES, pH 7.4). For indicator loading, bone slices prepared using a vibratome slicer were placed on glass-bottom dishes (Matsunami, Japan) and incubated in HEPES-buffered saline containing 15 μM Fura-2 AM (Dojindo, Japan) for 60 min at 37 °C. For ratiometric imaging, excitation wavelengths of 340 and 380 nm were alternately delivered, and an emission wavelength of >510 nm was detected by a cooled electron multiplying charge-coupled device camera (model C9100-13; Hamamatsu Photonics, Japan). The bone slices were mounted on an upright fluorescence microscope (DM6 FS, Leica) carrying a water immersion objective (HCX APO L 40×, Leica).

### Quantification and statistical analysis

All data obtained are presented as the means ± SEM. with n values indicating the number of examined mice or cells. Student *t*-test was used for two-group (Prism 7, GraphPad Software Inc.): *p* < 0.05 was considered to be statistically significant.

### Supplementary information

Supplemental Figure 1

Supplemental Figure 2

Supplemental Figure 3

Supplemental Figure 4

Supplemental Figure 5

Supplemental Table 1 and Supplemental Table 2

check list

original data files

## Data Availability

The original data of the article can be obtained from the corresponding author upon reasonable request. The raw data of microarray analyses have been deposited in the NCBI-GEO under accession numbers GSE105256 and GSE223776.
