# Supplementary figures and images for: Atypical cell death and insufficient matrix organization in long-bone growth plates from Tric-b-knockout mice
Source: Cell Death Dis. 2023 Dec 20;14(12):848. doi: 10.1038/s41419-023-06285-y (PMC10733378; doi:10.1038/s41419-023-06285-y)

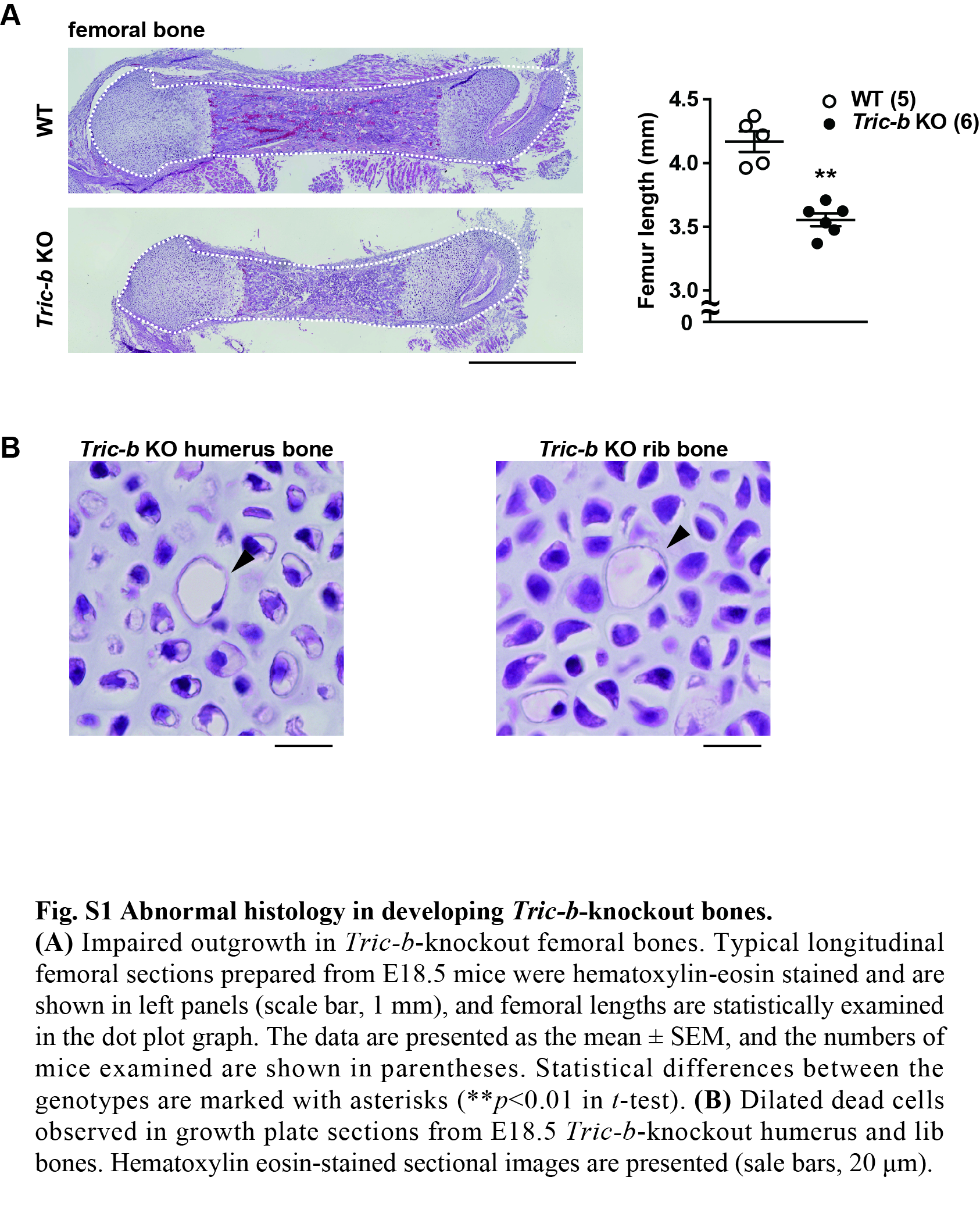

Supplement: Supplementary file 1 — Supplemental Figure 1 [file 41419_2023_6285_MOESM1_ESM.tif]

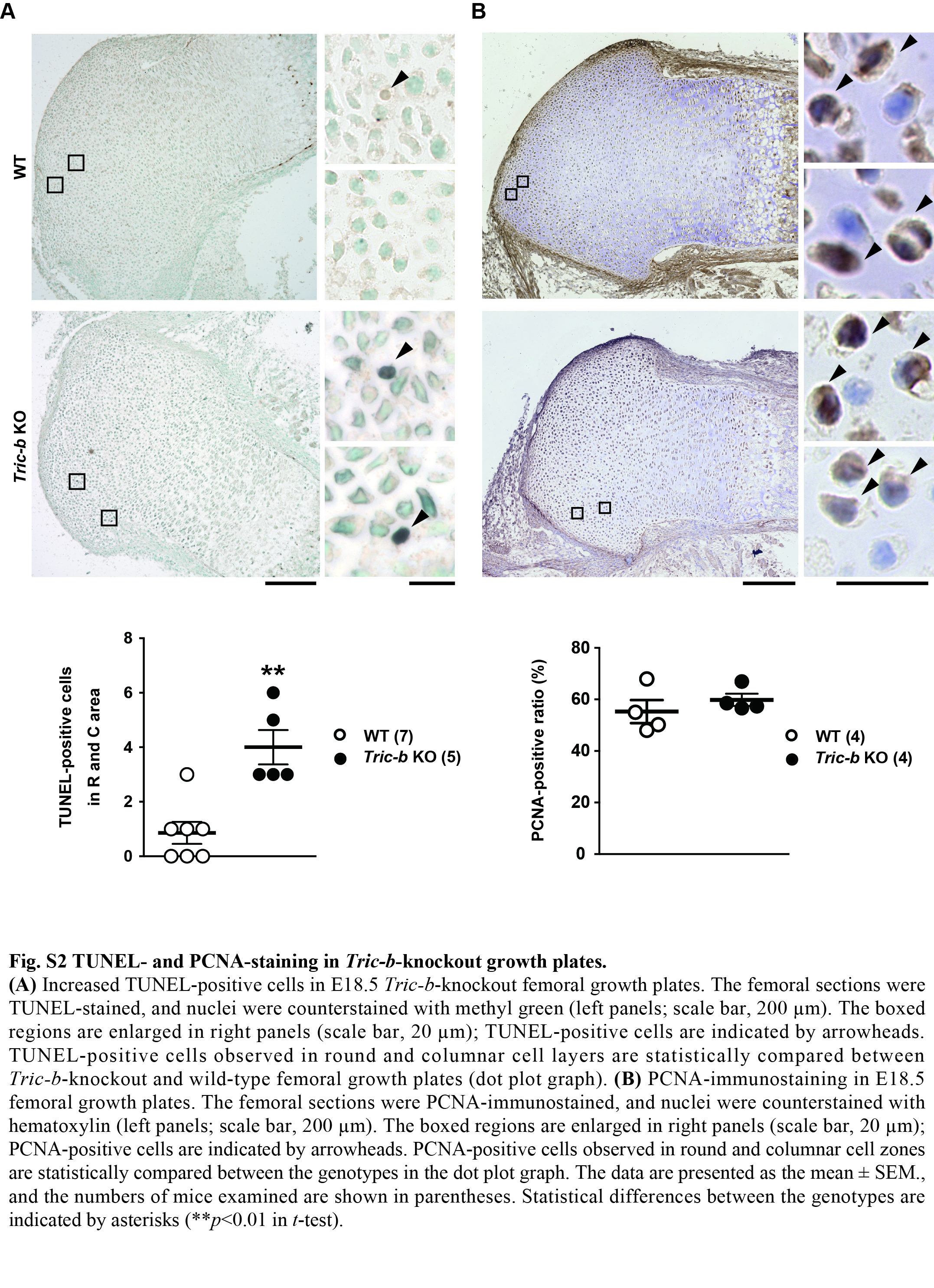

Supplement: Supplementary file 2 — Supplemental Figure 2 [file 41419_2023_6285_MOESM2_ESM.tif]

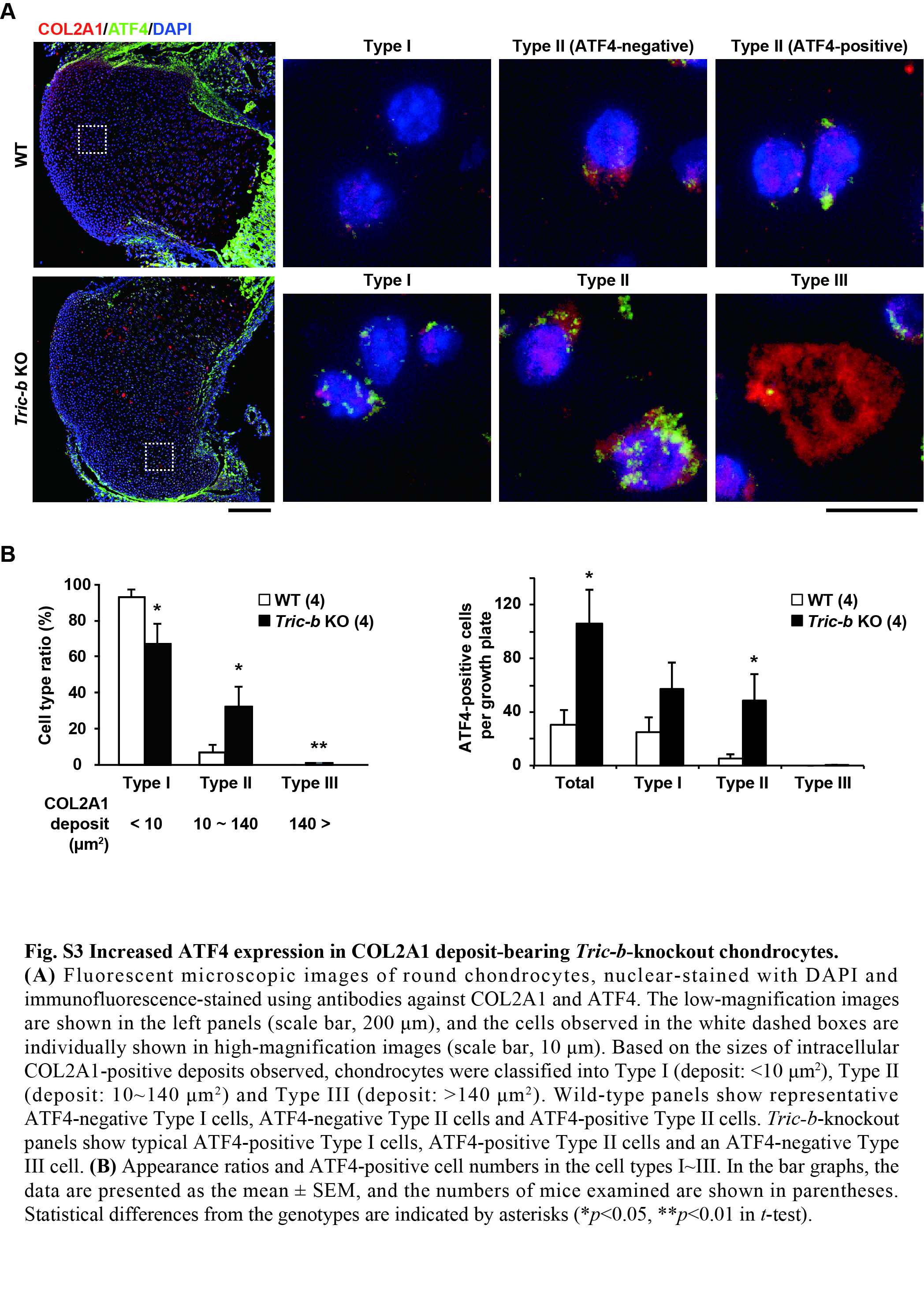

Supplement: Supplementary file 3 — Supplemental Figure 3 [file 41419_2023_6285_MOESM3_ESM.tif]

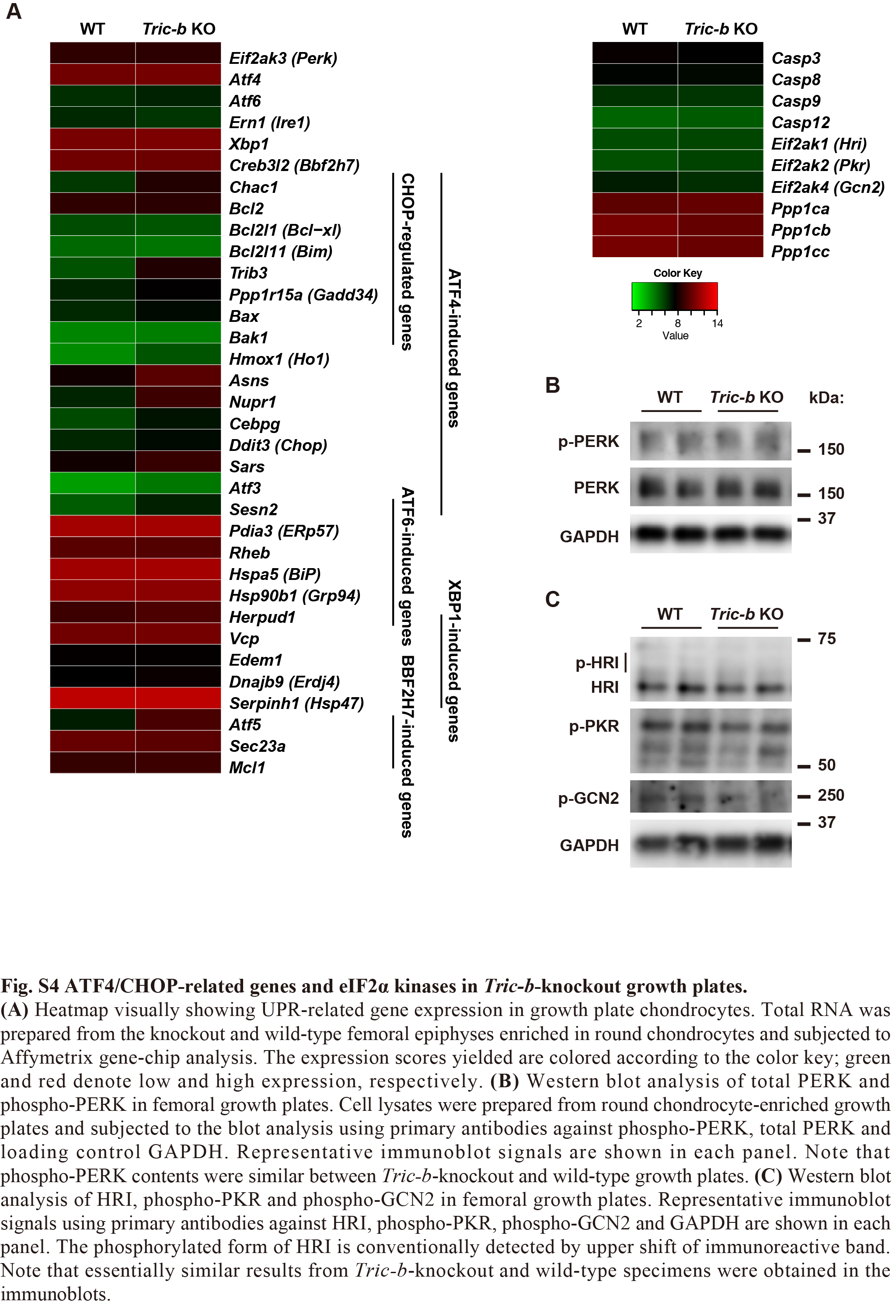

Supplement: Supplementary file 4 — Supplemental Figure 4 [file 41419_2023_6285_MOESM4_ESM.tif]

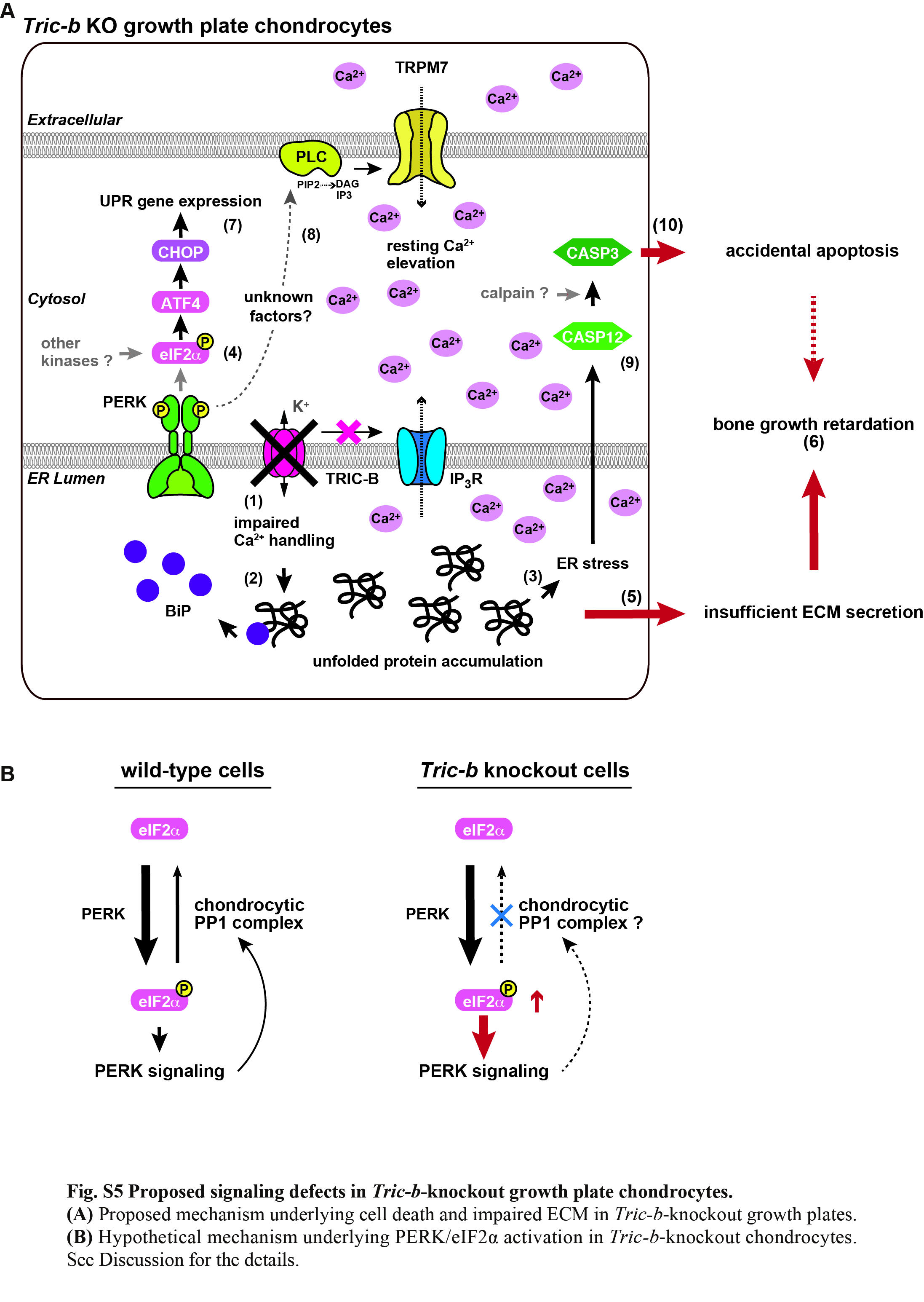

Supplement: Supplementary file 5 — Supplemental Figure 5 [file 41419_2023_6285_MOESM5_ESM.tif]
