# Supplementary material for: Atypical cell death and insufficient matrix organization in long-bone growth plates from Tric-b-knockout mice
Source: Cell Death Dis. 2023 Dec 20;14(12):848. doi: 10.1038/s41419-023-06285-y (PMC10733378; doi:10.1038/s41419-023-06285-y)
Supplement: Supplementary file 6 — Supplemental Table 1 and Supplemental Table 2 [file 41419_2023_6285_MOESM6_ESM.docx]

**Table S1　Antibodies used in this study.**

| **antigen** | **immunized animal & conjugation** | **source & catalogue number** |
| --- | --- | --- |
| COL2A1 | Rabbit, Unconjugated | ORIGENE, R1039 |
| KDEL | Mouse, Unconjugated | MBL, M181-3 |
| Mouse IgG (H+L) | Goat, Alexa Fluor 488 | Thermo Fisher Scientific, A-11029 |
| Rabbit IgG (H+L) | Goat, Alexa Fluor 555 | Thermo Fisher Scientific, A-21429 |
| ATF4 | Mouse, Unconjugated | Santa Cruz Biotechnology, sc-390063 |
| ATF6 | Mouse, Unconjugated | Cosmo Bio, BAM-73-505 |
| BiP | Mouse, Unconjugated | BD Transduction Laboratories, 610979 |
| Cleaved-CASP3 | Rabbit, Unconjugated | Cell Signaling Technologies, 9664 |
| CASP3 | Rabbit, Unconjugated | Cell Signaling Technologies, 14220 |
| CASP8 | Rabbit, Unconjugated | Cell Signaling Technologies, 4927 |
| Cleaved-CASP8 | Rabbit, Unconjugated | Novus, NB100-56116 |
| CASP9 | Rabbit, Unconjugated | Cell Signaling Technologies, 9504 |
| CASP12 | Rabbit, Unconjugated | Abcam, ab62484 |
| CHOP | Rabbit, Unconjugated | Cell Signaling Technologies, 5554 |
| Phospho-eIF2α | Rabbit, Unconjugated | Cell Signaling Technologies, 9721 |
| eIF2α | Mouse, Unconjugated | Santa Cruz Biotechnology, sc-133132 |
| BBF2H7 | Mouse, Unconjugated | Merck Millipore, MABE1018 |
| PERK | Rabbit, Unconjugated | Cell Signaling Technologies, 3192 |
| Phospho-PERK | Rabbit, Unconjugated | Cell Signaling Technologies, 3179 |
| HRI | Mouse, Unconjugated | Santa Cruz Biotechnology, sc-365239 |
| Phospho-PKR | Rabbit, Unconjugated | Abcam, ab32036 |
| Phospho-GCN2 | Rabbit, Unconjugated | Abcam, ab75836 |
| PCNA | Rabbit, Unconjugated | Abcam, ab18197 |
| GAPDH | Rabbit, Unconjugated | Sigma-Aldrich, G9545 |
| Rabbit IgG | Mouse, horseradish peroxidase | Santa Cruz Biotechnology, sc-2357 |
| mouse IgG | Rabbit, horseradish peroxidase | Dako, P0260 |

**Table S2　Primers used in this study.**

| **primer** | **forward** | **reverse** |
| --- | --- | --- |
| *Tric-b* | GTCATGGAGTACCCGTGGGATGA | TCTCCTGGTTCAACACGCTAGC |
| *Tric-b* |  | TTCATTCAGGGCACCGGACAGG |
| *Chak1* | CCCATTGCTGCTGCCAAT TC | GGCAGGTGATACTACCACGG |
| *Bcl2* | CTCTCGTCGCTACCGTCGTG | ACAATCCTCCCCCAGTTCAC |
| *Trib3* | CAACTGTGAGAGGACGAAGC | CACACCCAGGCTCCAGACAT |
| *Rheb* | TCCTCAGACATACTCCATAG | GTCCTTCTTATTTCCAACCA |
| *Ddit3 (Chop)* | CTGCCTTTCACCTTGGAGAC | CGTTTCCTGGGGATGAGATA |
| *Grp94* | TTGTGTCCAATTCAAGGTAATCA | TTGCTGACCCAAGAGGAAAC |
| *Herpud1 (Herp)* | GACCGCAGTTGGAGTGTGAG | ATGTCGCTTTTCCTTTGGAAGC |
| *Edem1* | AAGCCCTCTGGAACTTGCG | AACCCAATGGCCTGTCTGG |
| *Sec23a* | GCCCCACCACAACCTTAGCC | TTGAGTTTGAGCATCTGCCC |
| *Mcl1* | TCAAACAAAGAGGCTGGGATGG | ACATTTCTGATGCCGCCTTCT |
| *Casp3* | CTTCTTCAGAGGCGACTACT | TAAGCATACAGGAAGTCAGC |
| *Casp8* | AACTGTGTGACTCGCCAAGA | TCTGTTCCTTTTCTGTCCTT |
| *Casp9* | ACAAACTTGAGCACCGATTC | CACCACAAAGCAGTCCAGGG |
| *Casp12* | CTGATACTGACATTTTGAAC | AGGACTGGTGCTCTGGACGG |
| *Gapdh* | TGTGTCCGTCGTGGATCTGA | TTGCTGTTGAAGTCGCAGGAG |
| *Actb* | CATCCGTAAAGACCTCTATGCCAAC | ATGGAGCCACCGATCCACA |
| *Xbp1* | TGGCCGGGTCTGCTGAGTCCG | GTCCATGGGAAGATGTTCTGG |
