# Supplementary material for: Atypical cell death and insufficient matrix organization in long-bone growth plates from Tric-b-knockout mice
Source: Cell Death Dis. 2023 Dec 20;14(12):848. doi: 10.1038/s41419-023-06285-y (PMC10733378; doi:10.1038/s41419-023-06285-y)
Supplement: Supplementary file 8 — original data files [file 41419_2023_6285_MOESM8_ESM.pdf]

Figure 2C

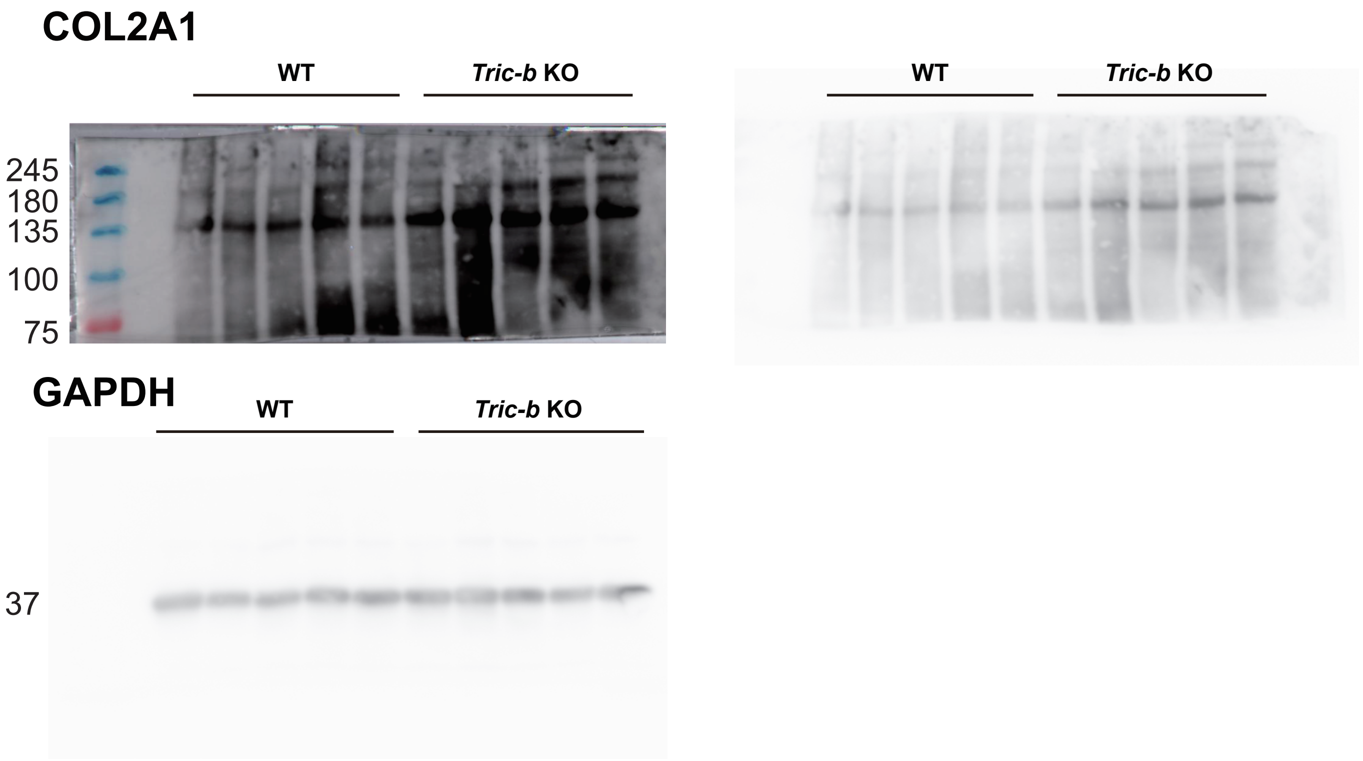

Figure 3A

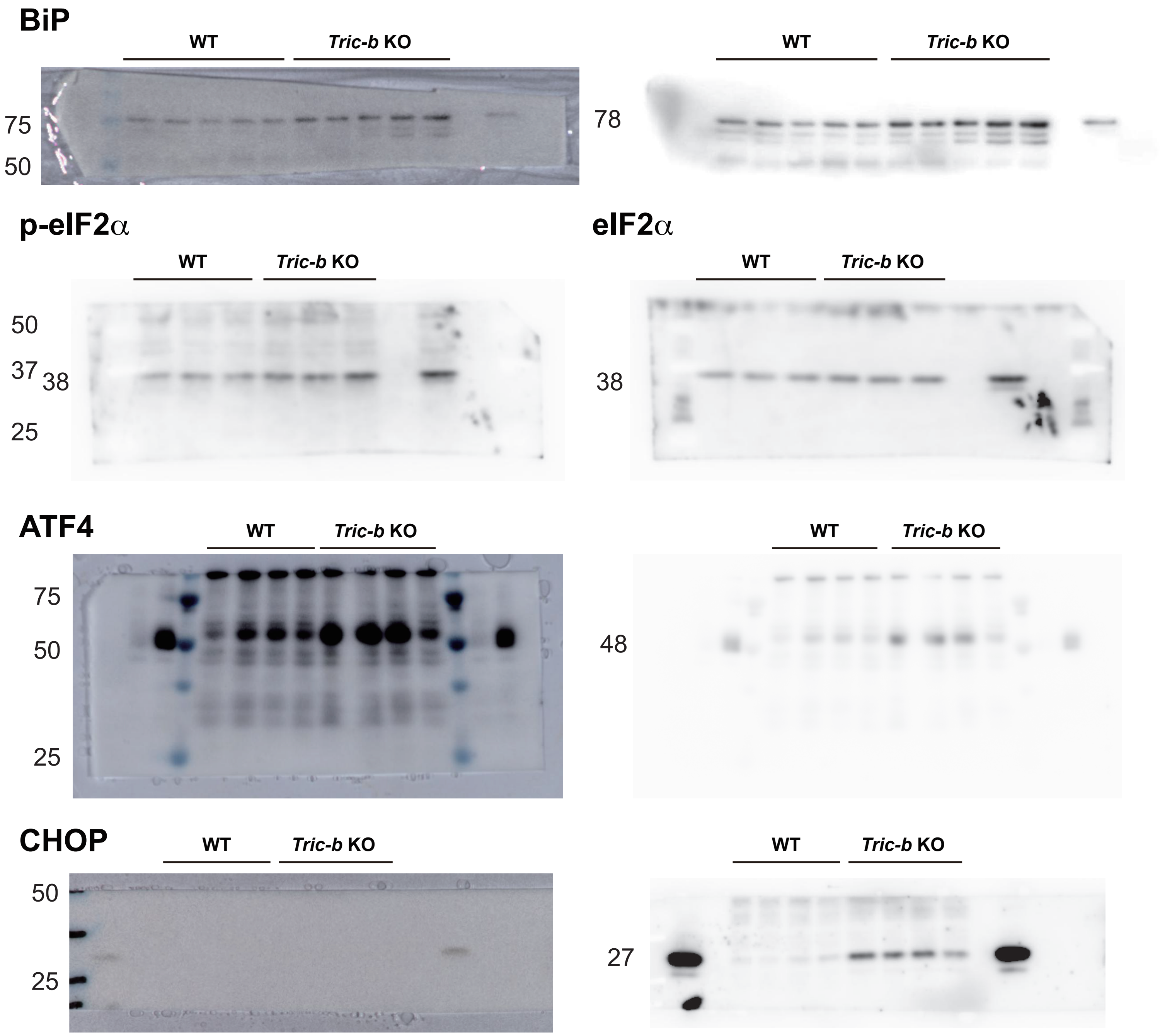

ATF6

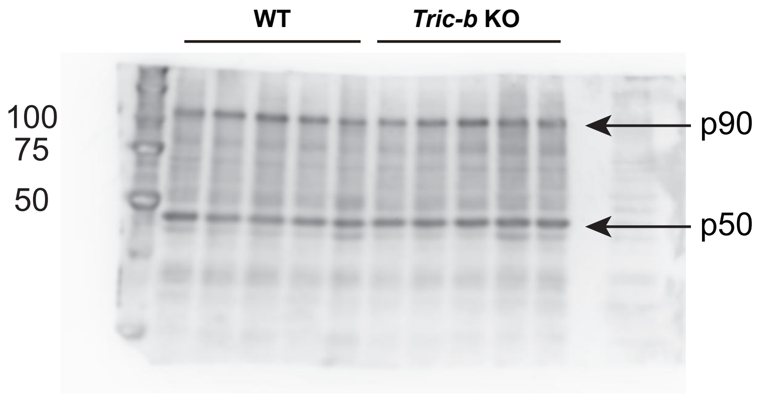

BBF2H7

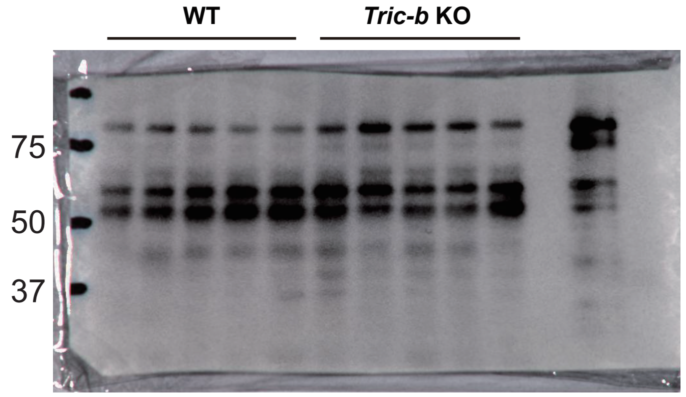

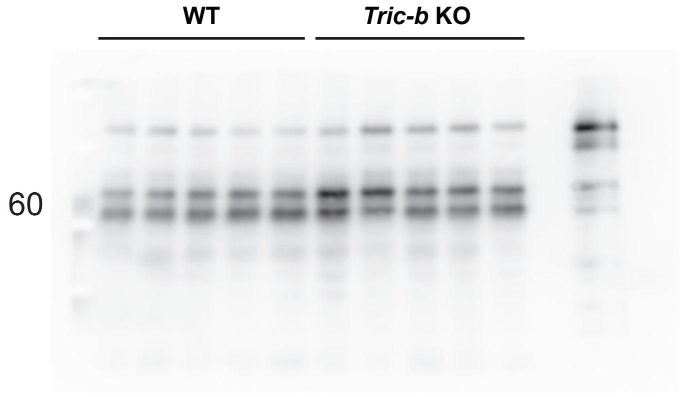

GAPDH

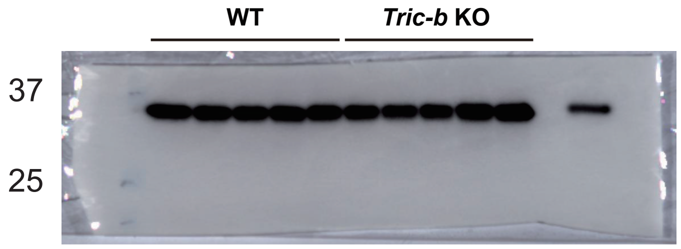

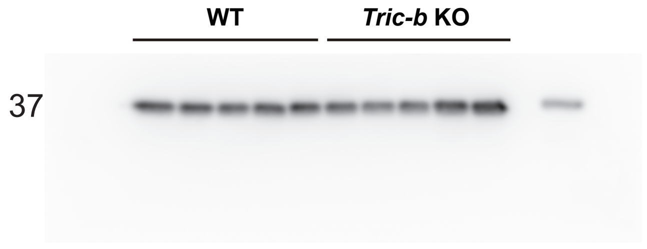

Figure 4A

Pro-CASP8

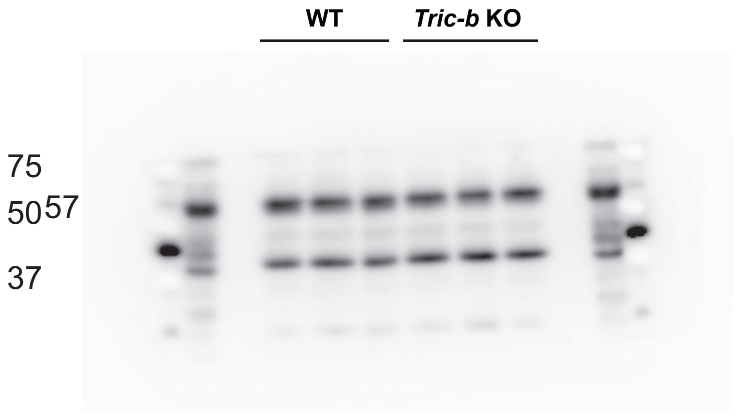

Cleaved-CASP8

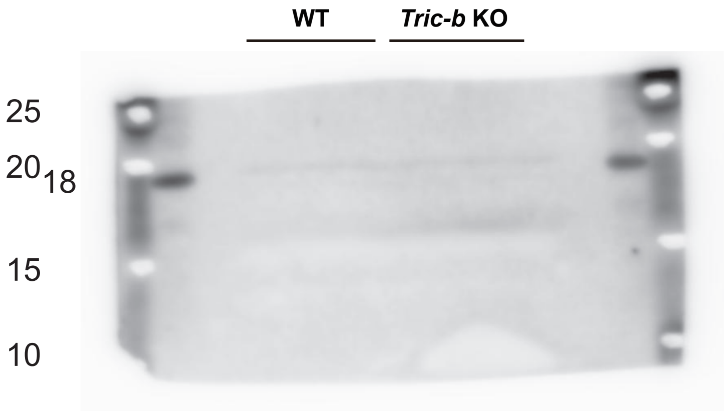

Pro-CASP9
Cleaved-CASP9

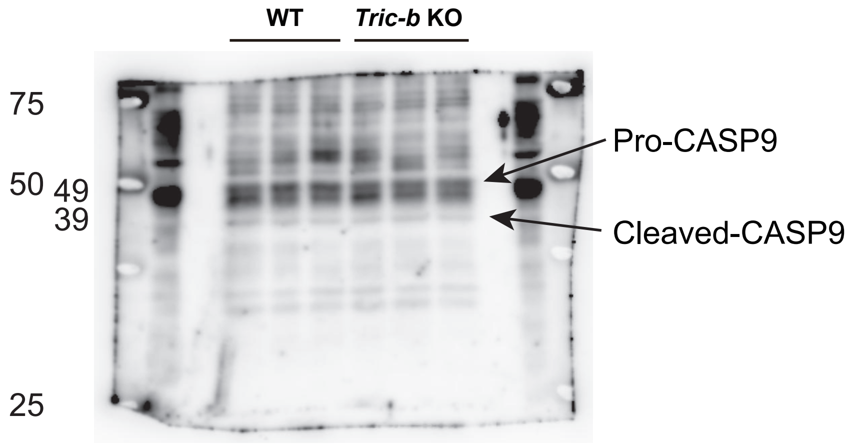

Pro-CASP12
Cleaved-CASP12

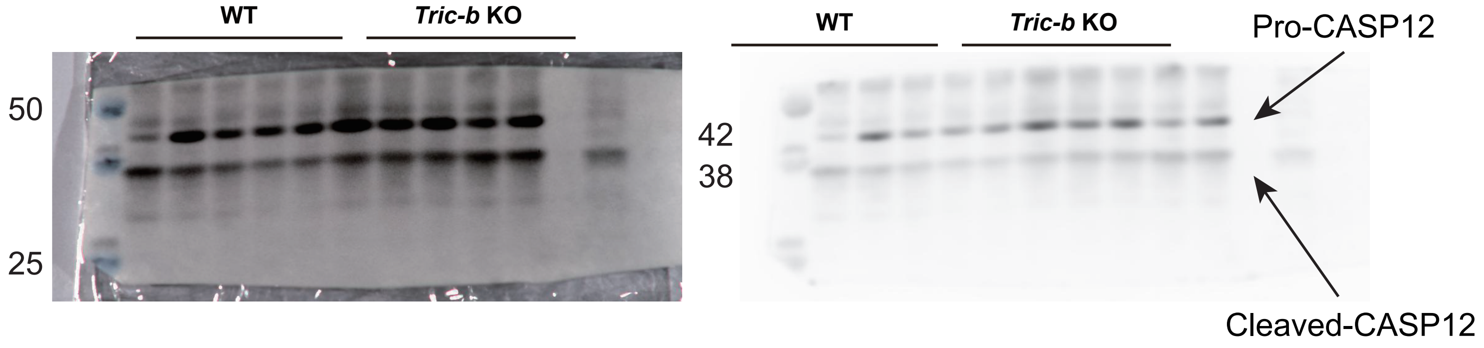

GAPDH

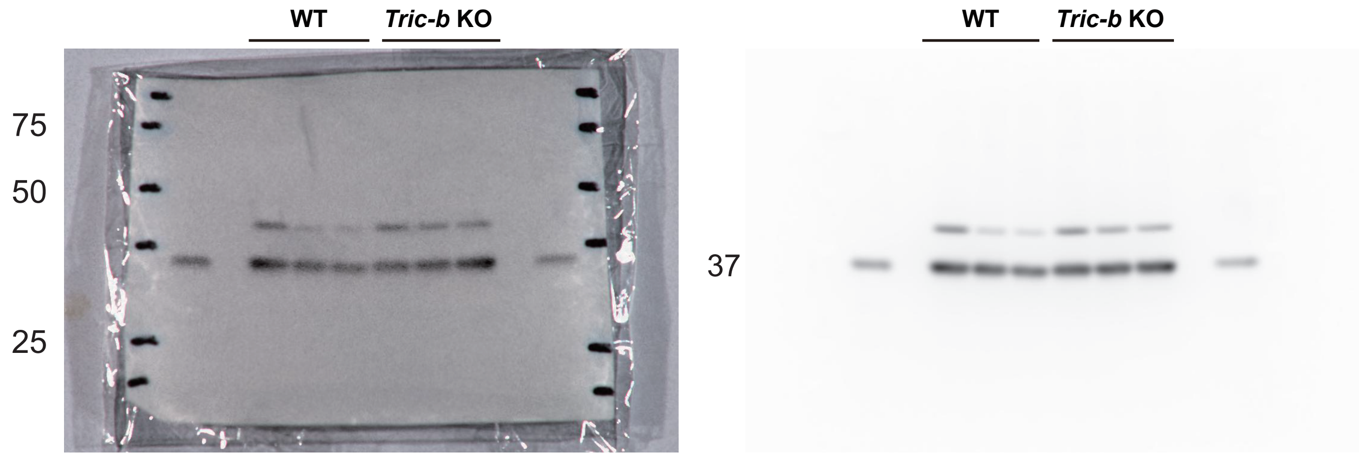

Figure 4B

Pro-CASP3

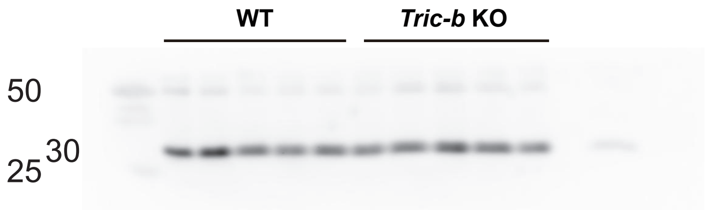

Cleaved-CASP3

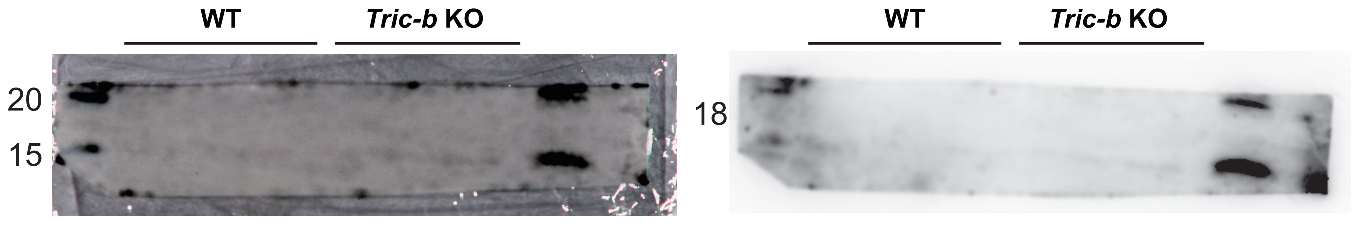

Figure S4B

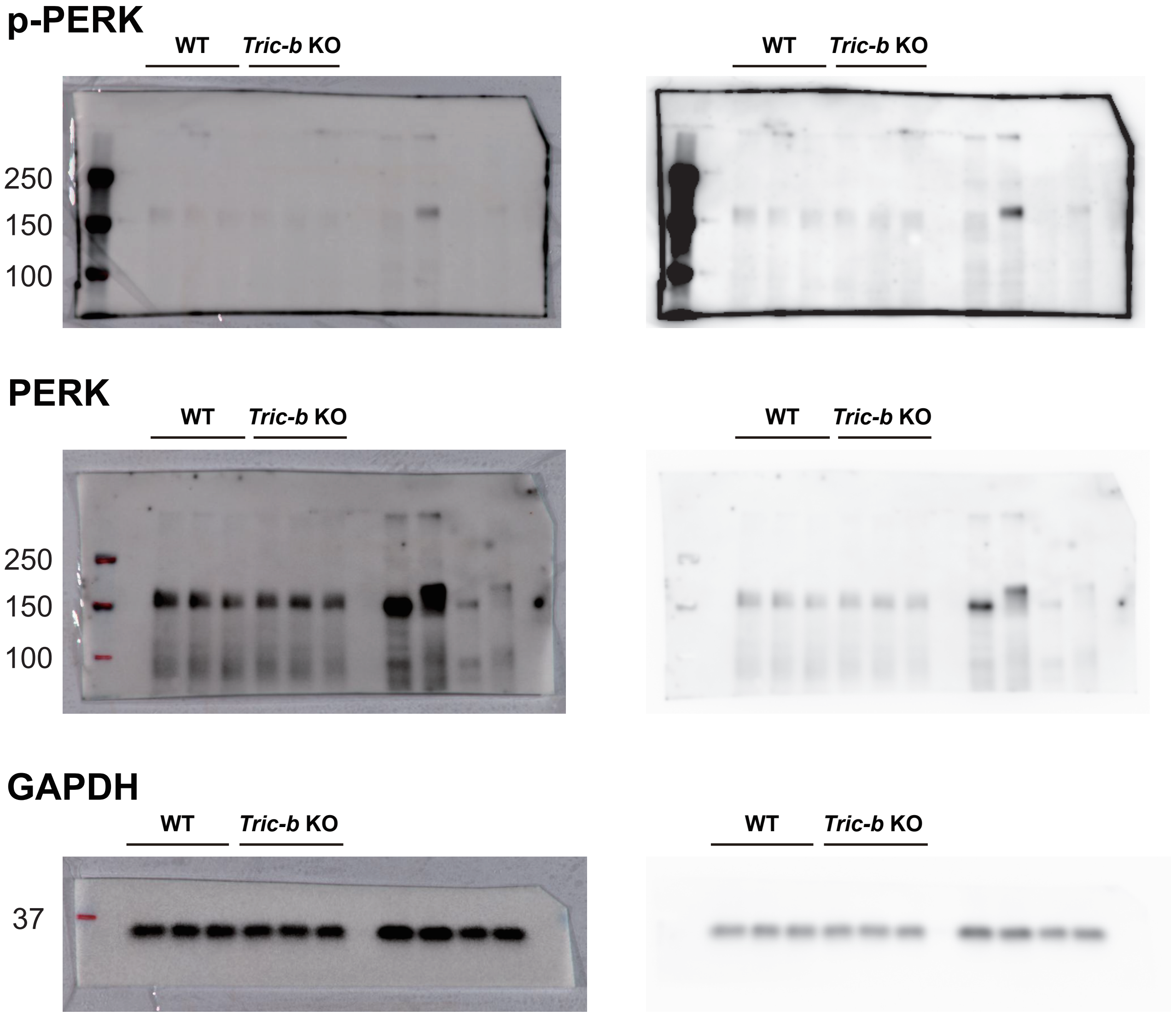

Figure S4C

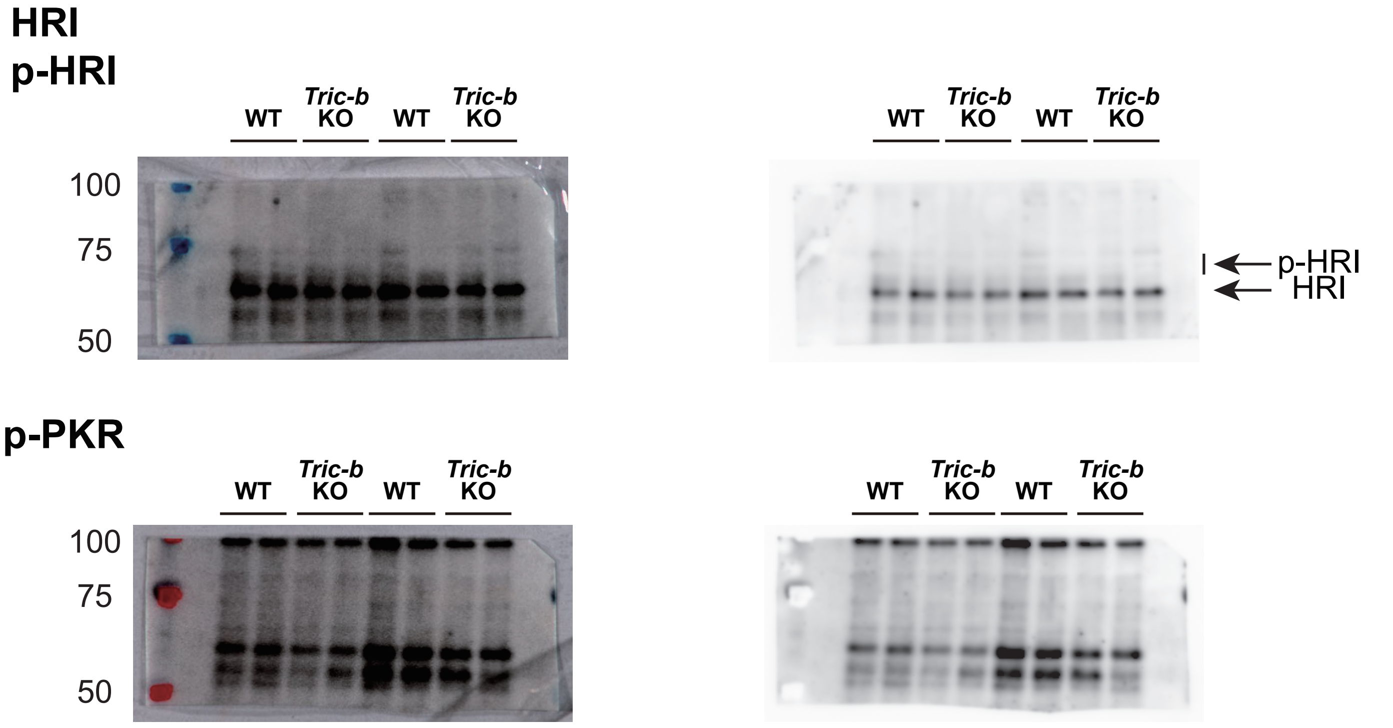

p-GCN2

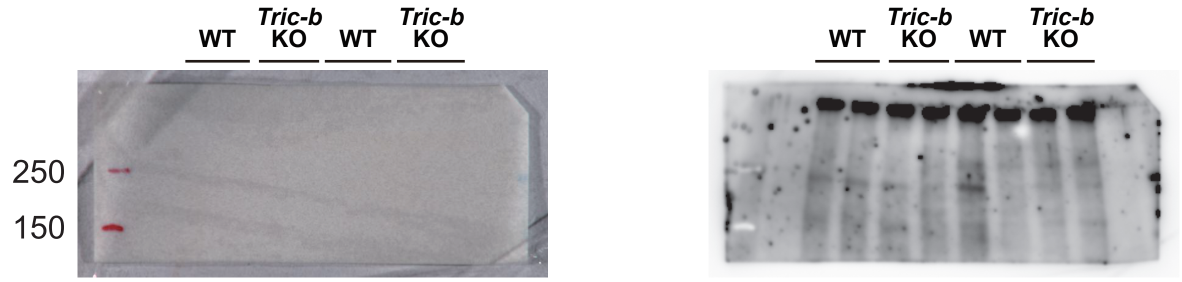
WT
Tric-b KO
WT
Tric-b KO
250
150
WT
Tric-b KO
WT
Tric-b KO

GAPDH

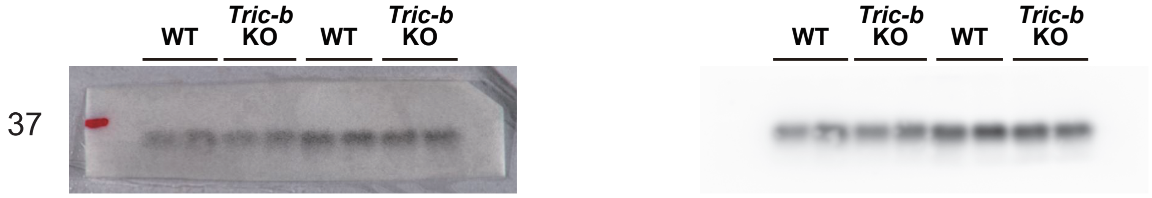
WT
Tric-b KO
WT
Tric-b KO
37
WT
Tric-b KO
WT
Tric-b KO
